# Assessing the impact of contraceptive use on mental health among women of reproductive age – a systematic review

**DOI:** 10.1186/s12884-024-06587-9

**Published:** 2024-05-30

**Authors:** Shayesteh Jahanfar, Julie Mortazavi, Amy Lapidow, Cassandra Cu, Jude Al Abosy, Katherine Morris, Juan Camilo Becerra-Mateus, Paola Andrenacci, Marwa Badawy, Meredith Steinfeldt, Olivia Maurer, Bohang Jiang, Moazzam Ali

**Affiliations:** 1 Department of Public Health and Community Medicine, Tufts University School of Medicine, Boston, US; 2 Universidad de Antioquia, Columbia, USA; 3 Coordinator Cochrane US Mentoring Program, Tufts University School of Medicine, Boston, US; 4 Cochrane mentee, US Mentoring Program, Tufts University School of Medicine, Boston, US; 5 Department of Sexual and Reproductive Health and Research, World Health Organization, Avenue Appia 20, Geneva 27, Geneva, CH-1211 Switzerland

**Keywords:** Contraception, Mental health, Depression, Anxiety, Systematic review

## Abstract

**Background:**

Contraceptive use is the principal method by which women avoid unintended pregnancy. An unintended pregnancy can induce long-term distress related to the medical, emotional, and social consequences of carrying that pregnancy to term.

**Objectives:**

This review investigates the effects of modern contraception techniques such as birth control pills, long-acting reversible contraceptives (e.g., intrauterine devices, implants), and condoms on mental health status.

**Methods:**

We searched multiple databases from inception until February 2022, with no geographical boundaries. RCTs underwent a quality assessment using the GRADE approach while the quality of observational studies was assessed using the Downs and Black scoring system. Data were analyzed through meta-analysis and relative risk and mean difference were calculated and forest plots were created for each outcome when two or more data points were eligible for analysis.

**Main results:**

The total number of included studies was 43. In women without previous mental disorders, both RCTs (3 studies, SMD 0.18, 95% CI [0.02, 0.34], high quality of evidence) and cohort studies (RR 1.04 95% CI [1.03, 1.04]) detected a slight increase in the risk of depression development. In women with previous mental disorders, both RCTs (9 studies, SMD − 0.15, 95% CI [-0.30, -0.00], high quality of evidence) and cohort studies (SMD − 0.26, 95% CI [-0.37, -0.15]) detected slight protective effects of depression development. It was also noticed that HC demonstrated protective effects for anxiety in both groups (SMD − 0.20, 95% CI [-0.40, -0.01]).

**Conclusions:**

Among women with pre-existing mental disorders who use hormonal contraceptives, we reported protective association with decreased depressive symptoms. However, the study also draws attention to some potential negative effects, including an increase in the risk of depression and antidepressant use among contraceptive users, a risk that is higher among women who use the hormonal IUD, implant, or patch/ring methods. Providers should select contraceptive methods taking individual aspects into account to maximize benefits and minimize risks.

**Supplementary Information:**

The online version contains supplementary material available at 10.1186/s12884-024-06587-9.

## Background

The utilization of contraception has experienced a significant upsurge among women globally. The number of women employing modern contraceptives has increased from 663 million to 851 million in the past two decades [1], and it is estimated that an additional 70 million women will utilize contraception by 2030 as access improves [1]. Modern contraceptive methods are categorized into short-acting, long-acting, and one-time barrier forms. Short-acting contraceptives, such as the pill (151 million users, 16%), injectables (74 million users, 8%), and patches and vaginal rings (less than 15 million users, less than 2%) [1], are widely used. Long-acting contraceptives, including intrauterine devices (159 million users, 17%), implants (23 million users, 2%), and female sterilization (219 million users, 24%) [1], are also popular. However, the prevalence of use for one-time barrier contraceptives, such as sponges, diaphragms, cervical caps, spermicide, female condoms, and male condoms, is low, except for male condom use (189 million users, 21%) [1].

Beyond preventing pregnancy, there are additional benefits to the use of contraception that are frequently overlooked. Evidence suggests that hormonal contraception has non-reproductive health advantages, including enhanced mental health status [2]. Within the United States, an estimated 1.5 million women use birth control pills for reasons other than pregnancy prevention, which has significant implications for women worldwide.

Hormonal fluctuations occur in women across various life stages, including puberty, menstrual cycles, pregnancy, and menopause, and these fluctuations of female ovarian hormones have a complex connection to mental health outcomes. The impact of modern contraception methods on the risk of adverse mental health outcomes such as depression, suicide, and anxiety is not yet clear. Depression and anxiety are among the most prevalent and disabling chronic diseases affecting reproductive-aged women globally, contributing to negative outcomes in reproductive health, including an increased risk of unintended pregnancy and its health and social consequences. Moreover, these conditions are precursors to numerous adverse perinatal and postpartum outcomes, including maternal and infant morbidity, obstetrical complications, preterm labor, stillbirth, low birth weight, and antepartum and postpartum depression. When pregnancy is unintended, the severity of these health events may be exacerbated [3]. Therefore, effective contraception plays a vital role in helping women who seek to prevent unintended pregnancy maintain a stable mental health status.

It is worth noting that studies are not immune from the “nocebo” effect. The nocebo effect is a psychological phenomenon in which the expectation of a negative outcome can itself contribute to negative outcomes. This can be observed when individuals anticipate or are told about possible side effects of a treatment, and as a result, they may experience those side effects even if the treatment is inert or harmless.

## Theoretical framework/ theory of change

**Fig. 1:**
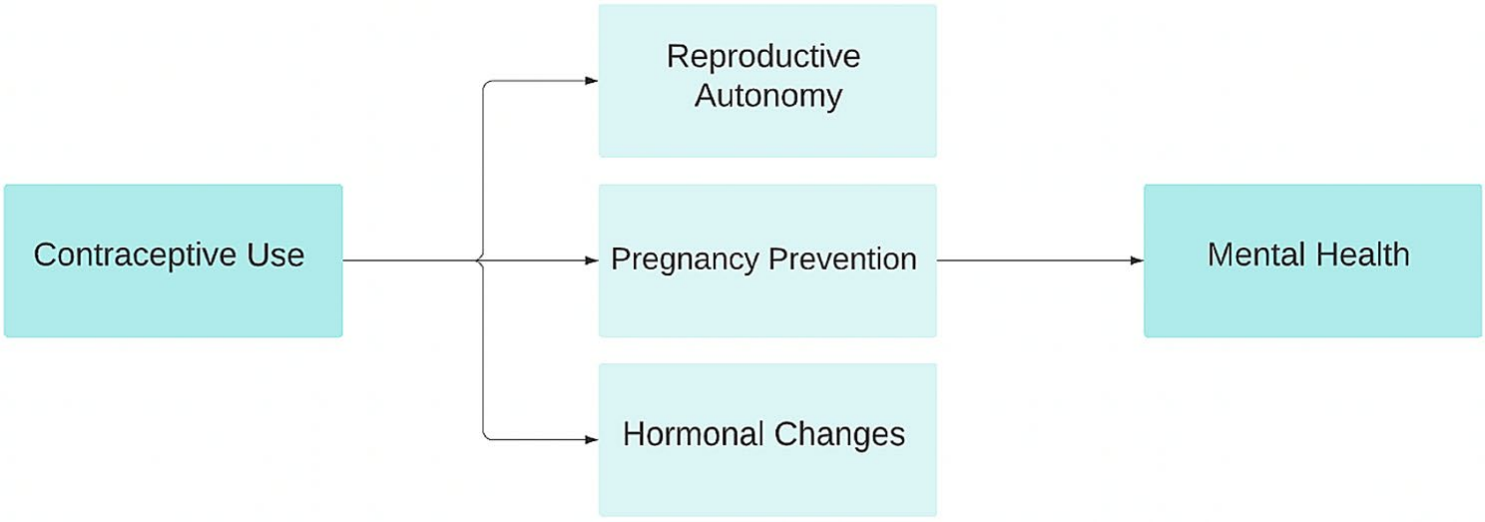
Family planning and its impact on mental health

Evidence suggests that modern contraception contributes to improved women’s health by reducing unintended and high-risk pregnancies, both of which can be stressful for any person [4]. Additionally, it is known that women who practice appropriate spacing of pregnancies and births (> 18 months) can focus more on their own physical and mental health as well as the health of other children and other family members [5]. Moreover, the impact of contraception on women’s socioeconomic status is well documented. Contraceptive use enables girls to remain in school for a longer period, leading to better occupation opportunities and empowering women economically. As a woman’s socioeconomic status improves, stressful economic events can be avoided [6]. For this reason, modern contraception is believed to improve the socioeconomic status of women as well as their dependents, which has positive effects on mental health status [7]. Additionally, lowered risk of unwanted pregnancy can increase enjoyment when engaging in sexual experiences with use of contraception can lead to improved quality of life and mental health [8] (See Fig. 1).

It is also important to consider the potential effects of contraceptive use on neurochemical and hormonal balance. It is widely agreed that depression and anxiety are impacted in part by deficiencies in neurotransmitters that affect mood [9, 10]. Conflicting research exists regarding whether a link exists between contraceptive use and neurotransmitter deficiency. One review reported no evidence for an association between the biochemical mechanisms of combined oral contraceptives (COC) and mood side effects reported by users [11]. Other prospective population-based cohort studies report similar or even lower rates of depression or mood symptoms in COC users when compared to nonusers [12]. Many of these recent studies have relied upon observational and cross-sectional designs and small sample sizes, so more research is needed that utilizes prospective, longitudinal, and randomized controlled trial designs to provide a more definitive assessment of the effects of contraception and mental health.

Contraceptives are currently recommended as part of a treatment for premenstrual dysphoric disorder, a subtype of depression [13]. Premenstrual dysphoric disorder is a time-limited and hormone-linked depression. Neurotransmitters, particularly gamma-aminobutyric acid and serotonin, appear to be linked with the manifestations of pre-menstrual disorder and premenstrual dysphoric disorder [14]. Research shows that lower levels of gamma-aminobutyric acid circulate during the luteal phase of the menstrual cycle [15]. This may help explain the benefit of combined hormonal contraceptives in treating premenstrual dysphoric disorder. However, some research suggests that progesterone’s involvement in the etiology of depression increases a woman’s risk for the use of antidepressants and a diagnosis of depression [16]. Therefore, it is important to create an accurate depiction of existing research on contraceptive use as it relates to mental health and highlight areas for future analysis.

It is also critical to focus on women of reproductive age as a population of interest. When compared to men, American women are more likely to experience a depressive or anxiety disorder [17]. This is an issue affected by intersectionality, as low-income, underinsured, and minority women are at an increased risk for both mental health disorders and adverse reproductive outcomes. Some research suggests that risk assessment, planning, social learning, decreased motivation and desire for self-care, excessive worry, and diminished perceptions of susceptibility to pregnancy may impact cognition and lead to suboptimal contraceptive choices among women with depression and anxiety [18]. Research has also shown that unilateral or bilateral oophorectomy can increase a woman’s overall risk of depression [19], due to substantial drops in estrogen production. It is important to utilize this information so that women seeking contraceptive methods or reproductive care can make the best possible decisions according to their specific pre-existing conditions and needs. This study aims to identify and evaluate evidence that focuses on the use of contraceptives and their impact on mental health status among women of reproductive age.

## Methods

This study is a registered systematic review of Prospero (CRD42022332647) and aims to examine the quantitative evidence regarding the use of contraceptives and their impact on mental health outcomes among women of reproductive age. The study adheres to the Population, Intervention, Comparison, Outcome, and Study design framework. The inclusion criteria specified that the study population should comprise women of reproductive age (14–49 years) presenting to primary healthcare clinics. The intervention considered modern contraception methods as effective and acceptable methods [20]. Observational studies were included, with contraceptive use as the primary exposure. Studies that combined contraception with other medications or modalities were excluded. The comparison was no contraceptive use. The outcome of interest was any effect on mental health status, including mood disorders such as depression, bipolar, and anxiety disorders, and psychotic disorders such as schizophrenia and post-traumatic stress disorder (PTSD). The study designs included were parallel or cluster randomized controlled trials, controlled clinical trials, controlled before and after studies, interrupted time series studies, cohort or longitudinal analyses, regression discontinuity designs, and case-control studies. A control group with no contraceptive usage was used to ensure that only studies with a comparison group were included.

To minimize publication bias, the study conducted a comprehensive search for published or unpublished studies from inception to February 2022 with no language or geographical boundaries. The search was performed in multiple databases, including CINAHL (1981–2022), OVID Medline (1946–2022), EMBASE (1947–2022), Psycho INFO (the 1800s-2022), Maternity & Infant Care (1857–2022), LILACS (1982–2022), clinical trial.gov (2000–2022), web of science (1900–2022), SCOPUS (2004–2022), and CENTRAL (1996–2022). Local databases of the World Health Organization (WHO) in various regions were also included in the search. We included WHO local databases as follows: Africa (AIM), Latin America and the Caribbean (LILACS), A network of Health Science Libraries across Asia (HELLIR), Virtual Health Sciences Library, IBECS (ibecs.isciii.es), SciELO (Scientific Electronic Library Online; www.scielo.br), LILACS (Latin American and Caribbean Health Sciences Literature; lilacs.bvsalud.org/en), PAHO (Pan American Health Library; www1.paho.org/english/DD/IKM/LI/library.htm), WHOLIS (WHO Library; dosei.who.int), WPRO (Western Pacific Region Index Medicus; www.wprim.org), Index Medicus for the South-East Asia Region (IMSEAR; imsear.hellis.org), IndMED (Indian medical journals; indmed.nic.in; 1985 onwards), Native Health Research Database (hscssl.unm.edu/nhd/).

In the inception phase of this systematic review, the inclusion criteria were initially limited to randomized controlled trials (RCT), but due to a paucity of available studies, quasi-experimental and observational studies, specifically cohort and case-control studies, were also incorporated. We used the Cochrane quality assessment (with Seven domains including selection bias, performance bias, detection bias, attrition bias, reporting bias, and other biases. This systematic review was done before changes were made in the Cochrane assessment to five domains.) for RCTs and Dawn and Black scale (with 27 questions relating to the quality of reporting (ten questions), external validity (three questions), internal validity (bias and confounding) (13 questions), and statistical power (one question) to assess the quality of observational studies. A GRADE table of summary of findings (incorporating the elements of risk of bias, inconsistency, indirectness, imprecision, and publication bias), was prepared for RCTs. All included articles were examined for a risk of bias using the critical appraisal checklists developed by the Cochrane Collaboration. The data extraction process was intended to be conducted by two independent reviewers, but due to resource constraints, only one reviewer was responsible for the extraction, with the other conducting checks. The targeted population in the protocol consisted of women of reproductive age (15–49 years), although several large cohort studies included both women of reproductive age and a small number of postmenopausal women whose data could not be separated. As a result, data from a few postmenopausal women were included in the review. Initially, the plan was to contact the primary authors of the studies to request clarification or obtain missing data, but time constraints precluded this approach. The inclusion criteria were initially limited to English-language studies, but the decision was made to expand the search to include studies in all languages to mitigate language-based bias in study selection. Non-English studies were translated into English. (See Appendix 1 for search strategy, and data sources).

We conducted a meta-analysis when we had two data points or more for each comparison and each outcome. Studies were combined for meta-analysis only when identical family planning devices/tools/drugs, dosages, and regimens were compared. Odds ratios (OR) or mean differences (MD) with a 95% confidence interval (95%CI) were calculated for each dichotomous or continuous outcome, respectively. The characteristics of included studies were recorded in a table, including the name of the first author, year of publication, country or study, study setting (public/private or rural/urban), type of family planning, dosage of contraception (if applicable), route of administration (if available), type of outcome studies, and effect measures associated with each outcome. Heterogeneity was visually examined by comparing study designs, target populations, and primary outcome measures across included studies. The homogeneity of trials combined in a meta‐analysis was assessed using both fixed‐effect and random‐effects models. The classical measure of heterogeneity, Cochran’s Q, was calculated as the weighted sum of squared differences between individual study effects and the pooled effect across studies, with the weights being those used in the pooling method. Q was distributed as a chi-square statistic, and the alpha level was set at 0.10 since the Chi2 test for heterogeneity is a low-power test. The I^2^ score was then used to identify the magnitude of heterogeneity. Any score of I^2^ above 50% was investigated for the clinical and methodological diversity of the studies. Pooling data from studies that had different contraceptive methods (e.g., contraceptive pills and transdermal patches), different doses of the same method, or different criteria for defining morbidity was not done. Subgroup analysis was conducted using different types of contraception, dose, and route of administration when possible. Sensitivity analysis was planned based on the study quality. It was also employed to test the robustness of any results that appeared to be based on heterogeneous combinations by examining the effect of deleting each study. Finally, sensitivity analyses were conducted based on rates of loss to follow‐up, and studies that had rates of loss to follow‐up over 20% were excluded.

## Results

**Fig. 2:**
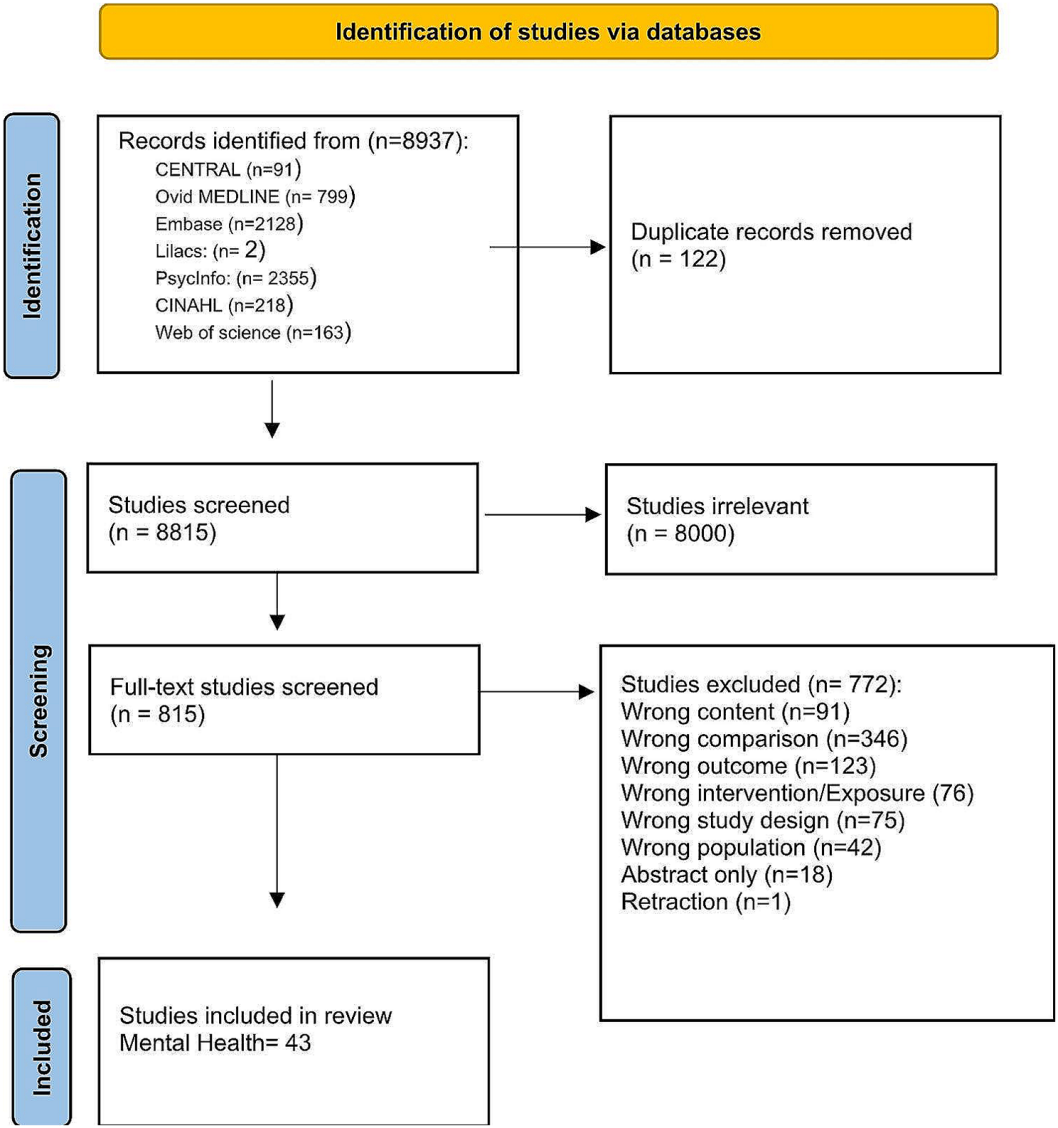
Flow diagram. From: Page MJ, McKenzie JE, Bossuyt PM, Boutron I, Hoffmann TC, Mulrow CD, et al. The PRISMA 2020 statement: an updated guideline for reporting systematic reviews. BMJ 2021;372:n71. doi: 10.1136/bmj.n71. For more information, visit: http://www.prisma-statement.org/

The Prisma chart in Fig. 2 demonstrates the number of studies included in the search from different sources as well as the number of studies screened and included in the review.

The total number of included studies was 43, 23 of which were RCTs and 20 of which were cohort studies. Table S1 (See Appendix 2) shows some of the characteristics of RCTs, including country of origin, year of publication, number of facilities, type of health facility, level of health facility, sample size, study design, population, type of contraception studied, the outcome of interest extracted, and quality of study based on study design. Similar data (with exposure instead of intervention) was extracted for observational studies (See Appendix 2, Table S2, and Table S3). Most of the studies were from 2000 onward, while a handful of studies were published before 2000 (*n* = 5).

Studies focused on either one contraception (oral contraception, ring/patch, implant, injection, intrauterine device (IUD), condoms, sterilization), a combination of contraceptives, or all hormonal contraceptives.

Comparisons were set based on available literature and the protocol on either all hormonal contraceptives versus no contraceptive use or oral contraceptive use versus no use. In cases where other types of contraceptives were studied, comparisons were made between use and non-use. Subgroup analysis can be seen in some of the forest plots where different contraceptives are used (pills versus IUDs, etc.)

Outcomes of interest included various aspects of mental illness, including depression, antidepressant use, anxiety, and suicide.

### Quality assessment

**Fig. 3:**
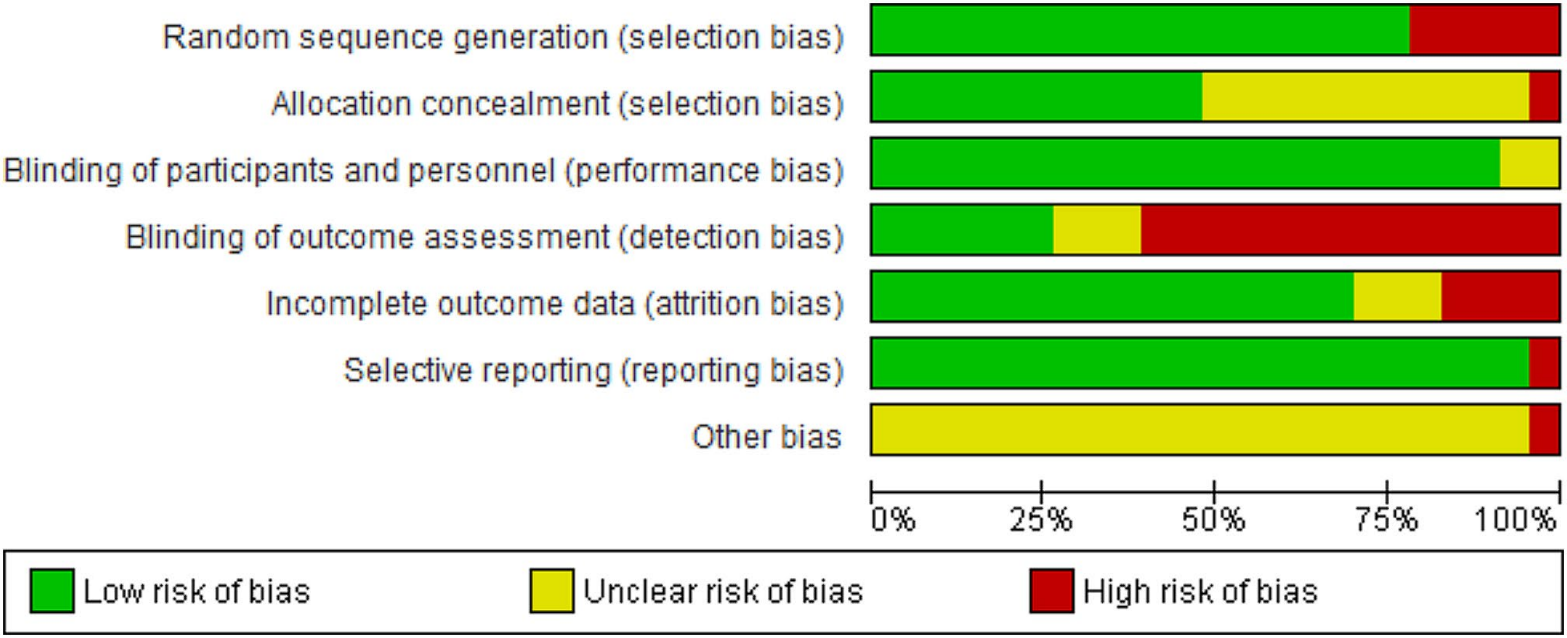
Risk of bias graph for included studies

Figure 3 presents the quality of assessment figures for RCT-included studies are presented below.

Table S4 shows the quality assessment of observational studies using the Black and Dawn scoring system. We considered the overall quality of evidence to be moderate for our review (mean: 13.65 ± 1.93, median = 14 min = 9, max = 16). Overall, we concluded that the quality of our evidence is moderate. (See appendix 2, Table S4)

#### Randomized clinical trials

**Fig. 4:**
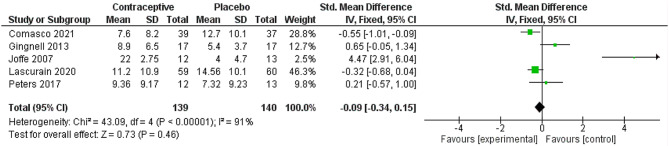
Forest plot on depression and OCP use for women with previous mental disorders in nine studies

The frequency of reporting this outcome and its clinical importance in non-reproductive health outcomes pertaining to oral contraceptive pill (OCP) use make this variable an attractive one to analyze. Depression was reported in nine studies for women with previous mental disorders, comparing OCP users with non-users [21–29]. The scales used to measure depression varied from comprehensive tools to depression as an item on a more elaborated tool like the Daily Record of Severity of Problems (DRSP). The meta-analysis showed a slightly protective difference between users and non-users of OCP in terms of depression in women with previous mental disorders (standardized mean differences (SMD): -0.09 (5 studies, high quality of evidence), 95% confidence interval (CI) -0.34, 0.15). Caution should be used in the interpretation of this analysis due to the high heterogeneity of 91%. (Fig. 4) The high level of heterogeneity did not change when we used random effect (-0.21 [-0.46, 0.04] with I^2^ of 89%) or run sensitivity analysis.

Since we had nine studies in this analysis, we created a funnel plot to assess the risk of publication bias. An asymmetric funnel plot showed publication bias is not likely (See Appendix 3, Figure S1).

We found five papers that reported depression as a continuous variable using the Montgomery-Asberg Depression Rating Scale [23, 25–28]. Hence, we reported these separately. The mean difference for these studies was protective as the mean difference was found to be -0.09 (95% CI -0.34, 0.15, high quality of evidence). (See Appendix 3, Figure S2).

In three studies involving women without previous mental disorders, depression was reported as a continuous variable, and OCP users were compared with non-users. The mean difference for these studies did not indicate a protective effect, as the mean difference was calculated to be 0.18 (95% CI 0.02, 0.34, high quality of evidence). (See Appendix 3, Figure S3).

Table S5 shows the GRADE assessment of the above-noted papers. All three variables discussed had moderate to high-quality evidence.

Cohort studies.

### Depression

**Fig. 5:**
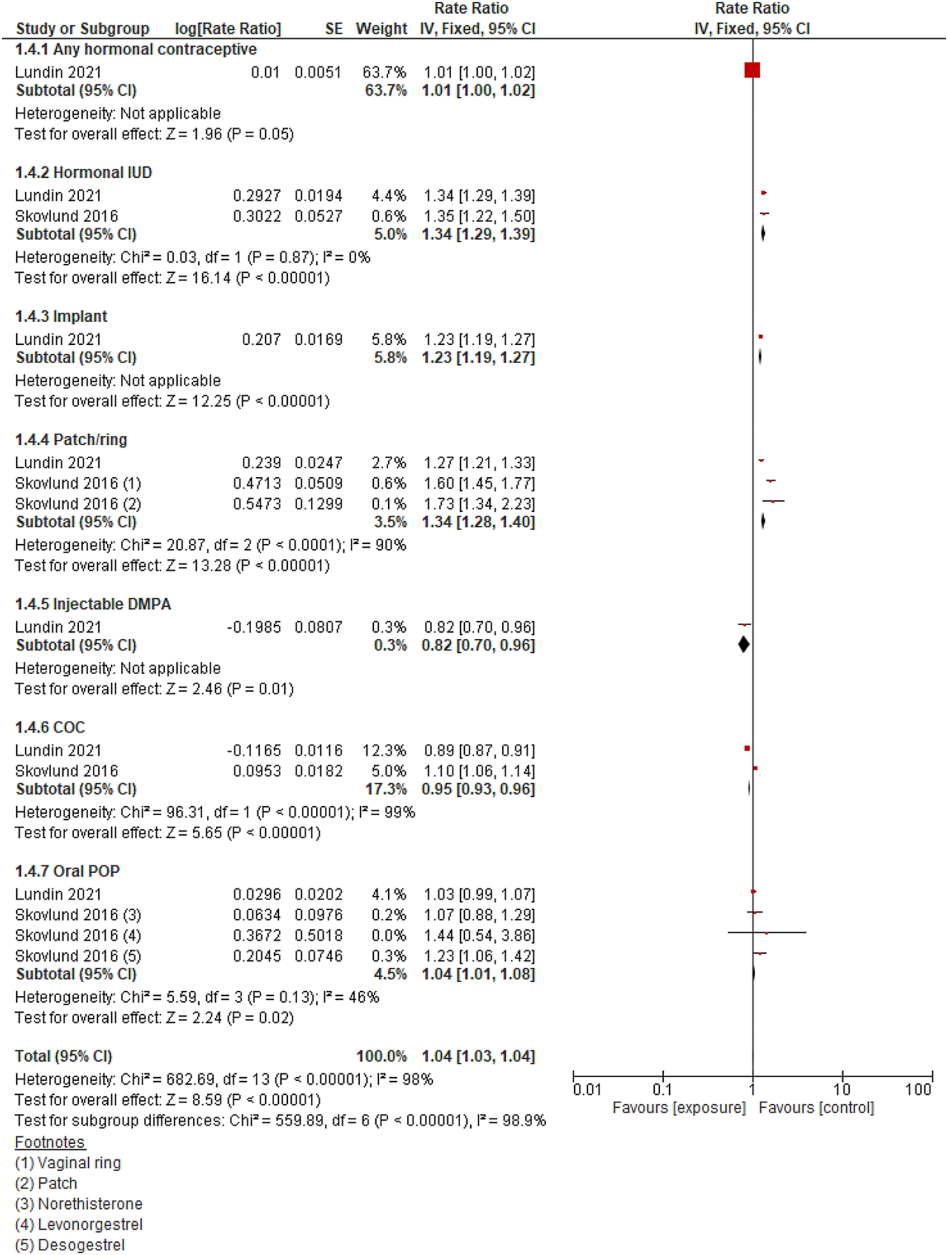
Use of hormonal contraceptives versus non-use for the dichotomous outcome of depression in women without previous mental disorders

The risk ratio for depression was found to be 1.13 (95% CI 1.04–1.24) among all hormonal contraceptive users compared with non-users. This analysis contained women without previous mental disorders reported by two large studies [16, 30]. The risk is similarly reported to be significantly higher on the user side for implants, progesterone pills/patches/rings, and hormonal IUDs. Heterogeneity was high (I^2^ = 99%). (Fig. 5)

Use of antidepressants was reported in 3 studies [31–33] with a total sample size of over one million and the number of events reported as around 30,000 incidents in women without previous mental disorders or general women. When all hormonal contraceptives (HC) were compared with no contraceptive use, the overall effect size was 45% higher among users compared to non-users (1.45, 95% CI 1.41, 1.49). The risk ratio from one study [30] for oral contraceptive users only was 0.89 (95% CI 0.55–1.44), demonstrating a non-significant but protective effect. Heterogeneity was high (I^2^ = 78.7%). This could be due to clinical heterogeneity (different study settings, type of contraception, etc.). However, the number of studies was low, not allowing for further investigation. (See Appendix 3, Figure S4).

Some studies [34–35] reported depression scores as a continuous variable using various tools (DRSP and the Quick Inventory of Depressive Symptomatology-Self-Report) in women with previous mental disorders. The standardized mean difference for depression among all hormonal contraceptive users was − 0.26 (-0.37, -0.15), which suggests a protective effect. The heterogeneity is low in this analysis (I^2^ = 0%). (See Appendix 3, Figure S5)

**Fig. 6:**
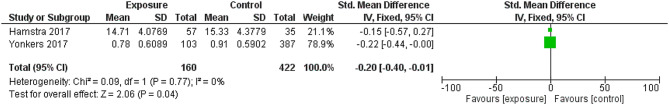
Use of hormonal contraceptives versus non-use for the continuous outcome of anxiety

For the outcome of anxiety, we found two studies. Anxiety score was reported using different tools. Yonkers [34] used DRSP with six levels, while Hamstra [36] utilized the Interpersonal Sensitivity Measure. We, therefore, used the standardized mean difference to analyze the data. The Hamstra study’s participants were Premenstrual Syndrome-free women, while the participants of the Yonkers study were women who sought treatment for Premenstrual Syndrome. There was a significant difference in the SMD of anxiety between hormonal contraceptive users compared with non-users (-0.20, [95% CI -0.40, -0.01]). In other words, HC users had 20% lower scores of anxiety compared to non-users. (Fig. 6)

The risk ratio for suicide was not significant among users of OCP compared with non-users in general women (1.17, [95% CI 0.96, 1.42]). The heterogeneity for this analysis was relatively high (I^2^ = 40%). (See Appendix 3, Figure S6)

We were unable to draw forest plots for other outcomes (suicide attempt as hazard ratio, risk of diagnosis of depression at 12 months, PTSD, anxiety as a dichotomous outcome, and number of major depressive episodes) but a summary of each study can be found in Appendices (See Appendix 2, Table S2).

## Discussion

### Summary of main results

Different results were observed regarding the association between HC use and mental health outcomes in women with and without previous mental disorders. In the meta-analysis of RCTs conducted on women with previous mental disorders, a slightly significant protective effect was observed, while the analysis on women without previous mental disorders showed a significant risk in depression scores. Cohort studies showed similar results for depression and antidepressant use. HC demonstrated protective effects for anxiety in both groups. OCP users did not show a significant risk of suicide compared to non-users. However, a subgroup analysis conducted by contraceptive type revealed that women who use hormonal IUD, implant, or patch/ring methods have a significantly higher risk of depression than other contraceptive methods. For oral contraceptive methods, varying results were observed, and the difference in effect on depression is not well understood when subgrouping by COC and POP types. Additionally, modification effects of age were observed across several studies. The risk of depression, antidepressants, or psychotropic drug use decreases as age increases from adolescence to adulthood.

#### Overall completeness and applicability of evidence

A noteworthy attribute of this review is the extensive search strategy employed, which encompassed several electronic databases and produced a diverse array of studies. However, the task of generating suitable recommendations concerning the utilization of contraceptives to enhance non-reproductive health outcomes in women is beset with difficulties arising from heterogeneity concerns.

#### Quality of evidence

In order to synthesize and communicate the findings pertaining to various variables, a Grades of Recommendation, Assessment, Development, and Evaluation evidence table was constructed for our RCT investigations, which reflected a high quality of evidence.

As the primary exposure in our review was contraceptive use, it was predominantly self-reported in most of the studies. The presence of recall and information biases represents a significant concern for investigations reliant on self-reported data on contraceptive use, as this may potentially lead to an underestimation of the true effect of contraceptive use on our targeted outcomes. In future research, recording techniques such as an on-time injection checklist or electronic pill count should be prioritized.

#### Agreements and disagreements with other studies

While our study identified negative mental health effects among women using contraceptives, it’s noteworthy to consider the contrasting findings from previous research. Schaffir’s study, for instance, reported either no effect or even a beneficial effect on mood among combined hormonal contraceptive users with no history of mental health issues [37]. In alignment with our study’s outcomes, Pérez-López’s investigation indicated a significantly higher risk of suicide among women treated with hormonal contraceptives [38]. Interestingly, a study on levonorgestrel intrauterine device users found no adverse effects on mental health, a result inconsistent with our findings, which indicated a higher risk of depression and increased antidepressant use among these users [39]. Moreover, this particular study highlighted a link between sexual dysfunction or low sexual function elevated depressive symptoms, and lower quality of life. It is worth noting that none of the studies explored the potential synergistic effect of sexual dysfunction and contraceptive use on mental health outcomes. Among women with pre-existing depressive or bipolar disorders who use hormonal contraceptives, we reported no association with increased depressive symptoms, similar to another review conducted [40]. Regrettably, none of the included studies investigated the association between contraceptives and the development of postpartum depression, highlighting a notable gap in the existing literature. These nuanced insights underscore the need for comprehensive research addressing the multifaceted relationship between contraceptive use and mental health outcomes, including potential synergies with sexual dysfunction and the postpartum period.

#### Strengths

Initially, a comprehensive search was conducted in the databases for all relevant studies, encompassing outdated as well as current contraceptive methods, with a wide scope of coverage. Subsequently, our investigations covered diseases and conditions that are commonly observed in women of reproductive age, for each outcome category. For a few of these outcomes, we identified a handful of studies that produced larger pooled sample sizes, resulting in enhanced statistical power, narrower confidence intervals, and more trustworthy findings. Furthermore, in addition to exploring the efficacy of contraception for its intended contraceptive purposes, we also investigated its effectiveness as a treatment. This supplementary evidence supports the use of contraception as a treatment in clinical settings and justifies reasonable insurance coverage.

#### Limitations

One limitation is that the duration of contraception use varied across studies, which could be an explanation for the heterogeneity. RCTs are shorter in duration compared to cohort studies, so the long-term effects of HC use on depression in a controlled setting are not fully understood. There is a complex interrelation between female hormones, how they fluctuate throughout the menstrual cycle, and mental health outcomes. Our study did not account for the menstrual cycle phase and how it interacts with contraceptives. Another limitation is the varying HC dosages and methods used across studies. We did not perform subgroup analysis on HC methods among our RCT studies, so we are unable to distinguish which drug dosage range and method is most effective at reducing or preventing mental health outcome risks. Time to depression occurrence and other mental health outcomes were not considered. Most of the included studies were on OCP users, with limited studies looking at the associations of long-term contraceptive types. As long-term contraceptive methods become more popular, it is essential to have more information on the long-term side effects of these methods. Another important limitation to mention is the lack of understanding we have about the context of the women’s lives who were included in the study. Studies greatly varied on important demographic risk factors collected such as parity, smoking and alcohol use, socioeconomic status, employment status, contraception history, etc. Varying scales were used, bringing into question the validity of the various tools used to measure the outcome of interest. Due to a lack of a common definition of mental health outcomes, bias in outcome measurement is a concern. Heterogeneity was a significant issue in this study, limiting our ability to investigate contraceptives’ effects further. Heterogeneity could be a result of variability in the population samples and definitions used to diagnose depression. To further investigate this issue, subgroup analysis should be performed to reduce the variability in the samples being compared across studies. For instance, some studies were conducted among healthy women, while some were conducted among women with existing mental health conditions, or among women serving in the military. Issues with randomization showed imbalances in intervention vs. placebo groups within several studies, showing that the intervention group had higher proportions of women with depression assigned to them compared to the control group. Some of the findings may also be limited to generalizability due to homogenous samples.

In cohort studies relying solely on registry data, the concept of a “large false negative population in the control group” underscores a significant limitation. This means that within the group designated as the control—individuals not exposed to a specific intervention or condition—there is a notable number of cases where the actual presence of the condition or outcome under investigation is inaccurately recorded in the registry data. This introduces a risk of misclassification and could lead to an underestimation of the true prevalence of the condition in the control group. Furthermore, the term “likely selective prescribing of LNG IUD in the depression group” highlights another potential challenge. It suggests a bias in the prescription patterns of the levonorgestrel intrauterine device (LNG IUD) toward individuals with depression. In other words, those with depression might be more likely to receive the LNG IUD compared to individuals without depression. This introduces a source of bias, as the intervention is not randomly assigned but rather influenced by the presence of a specific condition, potentially impacting the study’s internal validity and generalizability of findings.

The absence of contraceptive usage in a control group warrants careful consideration and discussion in research, as it introduces potential complexities and differences within the study population. The question of why individuals in the control group are not using contraception is crucial, as it can signify various factors that may influence the study outcomes.

The decision not to use contraception could stem from factors such as a lack of perceived need, personal beliefs, cultural considerations, access barriers, or fertility-related intentions. Each of these reasons introduces inherent differences within the control group, making it inherently distinct from individuals who actively choose or require contraception.

This divergence in baseline characteristics can pose challenges in isolating the specific effects of the contraceptive method under investigation. The comparison between a group actively using contraception and another not using any introduces confounding variables, potentially clouding the interpretation of results. Researchers need to thoroughly explore and discuss these differences to provide a comprehensive understanding of the study population and to acknowledge potential sources of bias.

#### Implications for practice

The results of our research shed light on the complex relationship between hormonal contraception (HC) use and mental health outcomes among women, revealing divergent findings based on previous mental health status and contraceptive methods. While a slightly significant protective effect of HC was observed in women with previous mental disorders, a significant risk in depression scores was noted among women without prior mental health issues. Interestingly, anxiety showed a protective effect across both groups. Notably, subgroup analysis highlighted a significantly higher risk of depression among users of hormonal IUDs, implants, or patches/rings compared to other methods. However, the distinction in the effect on depression between combined oral contraceptives (COC) and progestogen-only pills (POP) remains unclear. Moreover, age emerged as a modifying factor, with a decreasing risk of depression and antidepressant use observed as age increases from adolescence to adulthood. Despite the comprehensive search strategy employed, generating recommendations for contraceptive use to improve non-reproductive health outcomes in women remains challenging due to heterogeneity concerns. Nonetheless, our high-quality evidence underscores the importance of future research endeavors focusing on standardized measurement tools, exploring the contextual factors of contraceptive use, and investigating the long-term effects of various contraceptive methods on mental health outcomes. These findings provide valuable insights for clinicians, researchers, and stakeholders to optimize contraceptive selection and monitoring practices, ultimately promoting the holistic well-being of women.

#### Implications for research

The present study provides implications for subsequent research in this area. Our work suggests that different lengths of contraceptive use may have health effects in different directions and to different degrees. Therefore, the non-reproductive health benefits of different durations of contraceptive use should be further examined. Time-to-event analysis should be conducted to further understand the association between the start of contraception and the incidence of depression and other mental health outcomes. This study did not focus on the health effects of hormonal contraceptives with various components and doses. As more new contraceptive methods become available, research on the benefits of different hormonal components and doses on non-reproductive health may provide more guidance for clinical use. More studies on long-term modern contraception methods are needed as well as studies accounting for menstrual phases. To generalize findings to the larger population, future RCTs should use strict definitions of mental illness and selection criteria to ensure reproducibility across studies. Also important is the ability to understand the context of the women participating in these studies. Common predictors across studies should be used to properly account for confounders and track them. Additionally, future studies should consider the impacts of unintended pregnancy on postpartum women using contraceptives as well as the duration of contractive use and its impact on mental health.

### Electronic supplementary material

Below is the link to the electronic supplementary material.

Supplementary Material 1

Supplementary Material 2

Supplementary Material 3

Supplementary Material 4

## Data Availability

The datasets used and/or analyzed during the current study are available from the corresponding author upon reasonable request.

## Acronyms

COC: Combined oral contraceptive
CI: Confidence interval
DRSP: Daily Record of Severity of Problems
HC: Hormonal contraceptive
IUD: Intrauterine device
OC: Oral contraceptive
OCP: Oral contraceptive pill
OR: Odds ratio
PTSD: Post-traumatic stress disorder
RCT: Randomized controlled trial
SMD: Standardized mean differences
WHO: World Health Organization
