# Supplementary material for: Assessing the impact of contraceptive use on mental health among women of reproductive age – a systematic review
Source: BMC Pregnancy Childbirth. 2024 May 30;24:396. doi: 10.1186/s12884-024-06587-9 (PMC11137968; doi:10.1186/s12884-024-06587-9)
Supplement: Supplementary file 1 — Supplementary Material 1 [file 12884_2024_6587_MOESM1_ESM.docx]

**Appendix 1**

**Appendix *: Search strategy***

First, we will conduct a Medline search. The following medical subject heading (MeSH) search terms and keywords will be used, either independently or in combinations:

We will also consider adding the following terms for **family planning**.

"Family Planning Services"[Mesh] OR  "Family Planning Policy"[Mesh] OR  “planned pregnancy” [TW]  OR “planned pregnancies “[TW]  OR “one child policy” [TW]  OR “one child policies” [TW]  OR “pro natalist policy” [TW]  OR “pronatalist Policy” [TW]  OR  “pro natalist policies” [TW] OR “pronatalist Policies” [TW] OR “Antinatalist policy” [TW]  OR “Antinatalist policies” [TW]  OR"Contraception"[Mesh] OR "fertility inhibition"[TW]  OR "fertility control"[TW]  OR contraception* [TW] OR contraceptive*[TW]  OR Immunocontraception [TW] OR"Contraception Behavior"[Mesh] OR "contraceptive behavior" [TW] OR "contraceptive behaviour" [TW] OR "contraceptive behaviors" [TW] OR "contraceptive behaviours" [TW] OR “contraceptive usage” [TW] OR “contraceptive method switching” [TW] OR “Perinatal Care"[Mesh] OR "postnatal care" OR "postpartum care" OR “perinatal Care” [TW] OR ( postpartum [TW]  AND program*[tw]) OR "Birth Intervals"[Mesh] OR "birth interval" [TW] OR "birth intervals" [TW] OR "birth spacing" [TW] OR "pregnancy”, OR “DMPA”, OR “vaginal rings”, OR “modern contraception”.

The family planning terms will then be added to the following balloon search themes below.

Mesh terms for **mental health** are inclusive of:

“mood disorders” [MeSH] OR “anxiety” [MeSH] OR “depressive disorder” [MeSH] OR "depression" [MeSH] OR “mental health” [MeSH] OR “schizophrenia” [MeSH] OR “bipolar disorder” [MeSH] OR “stress disorders, post-traumatic” [MeSH] OR anxiety OR mood OR depression OR affective disorder OR mental health OR schizophrenia OR “bipolar disorder” OR PTSD

**Appendix : *Data sources***

**Global databases**

Cumulative Index to Nursing and Allied Health Literature (CINAHL)

<https://www.ebsco.com/products/research-databases/cinahl-database>

OVID Medline

<https://www.wolterskluwer.com/en/solutions/ovid/ovid-medline-901>

Embase

<https://www.embase.com>

PsycINFO

<https://www.apa.org/pubs/databases/psycinfo>

Maternity & Infant Care

<https://www.midirs.org/resources/maternity-and-infant-care-mic-database/>

Clinical trial.gov

<https://clinicaltrials.gov/>

Web of science

<https://clarivate.com/webofsciencegroup/solutions/web-of-science/>

Scopus

<https://www.scopus.com>

Cochrane Central Register of Controlled Trials (CENTRAL)

<https://www.cochranelibrary.com/central>

**Local databases**

Africa (AIM)

<http://indexmedicus.afro.who.int/>

Latin America and the Caribbean (LILACS)

<http://bases.bvs.br/>

A network of Health Science Libraries across Asia (HELLIR)

<http://www.hellis.org>

Virtual Health Sciences Library

<http://www.emro.who.int/HIS/VHSL/>

IBECS

<http://ibecs.isciii.es>

Scientific Electronic Library Online (SciELO)

<http://www.scielo.br>

Pan American Health Library (PAHO)

<https://www.paho.org>

WHO Library (WHOLIS)

<http://dosei.who.int>

Western Pacific Region Index Medicus (WPRO)

<http://www.wprim.org>

Index Medicus for the South‐East Asia Region (IMSEAR)

<http://imsear.hellis.org>

Indian medical journals (IndMED)

<http://indmed.nic.in>

Native Health Research Database

<http://hscssl.unm.edu/nhd/>

**Other databases**

International Union for the Scientific Study of Population (IUSSP)

<http://iussp.org/>

Population Association of America (PAA)

<https://www.populationassociation.org>

International Conference on Family Planning (ICFP)

<http://www.fpconference2013.org/>

Department for International Development (DFID)

<https://www.gov.uk>

Canadian funding for international development projects (CIDA)

<https://www.international.gc.ca>

U.S. Agency for International Development (USAID)

<https://www.usaid.gov>

**University databases for thesis and technical reports**

The London School of Hygiene & Tropical Medicine (LSHTM) Database

<https://www.lshtm.ac.uk/>

Harvard University Database

<https://library.harvard.edu>

University of California Berkley Database

<https://www.lib.berkeley.edu>

George Washington University Database

<https://library.gwu.edu>

**Websites of relevant societies and institutions**

World Health Organization (WHO)

<https://www.who.int>

Guttmacher institute

<https://www.guttmacher.org>

Department for International Development (DFID)

<https://www.gov.uk>

U.S. Agency for International Development (USAID)

<https://www.usaid.gov>

Marie Stopes International (MSI)

<https://www.msichoices.org/>

Population Services International (PSI)

<https://www.psi.org>

United Nations Population Fund (UNFPA)

<https://www.unfpa.org>

Population council

<https://www.popcouncil.org>
