# Supplementary material for: Assessing the impact of contraceptive use on mental health among women of reproductive age – a systematic review
Source: BMC Pregnancy Childbirth. 2024 May 30;24:396. doi: 10.1186/s12884-024-06587-9 (PMC11137968; doi:10.1186/s12884-024-06587-9)
Supplement: Supplementary file 2 — Supplementary Material 2 [file 12884_2024_6587_MOESM2_ESM.pdf]

Table S1: Characteristics of Randomized Controlled Trials

Study (year)	Country	Study setting (public /private or rural/urban)	Study design	Sample size	Study aim	Population	Outcome (s) of interest	Intervention (RCTs)	Period of observation (weeks, months, years)	Findings (effect size and 95%CI)	Note
Akhondzadeh 2003	Iran	Private	RCT	32	Investigate the efficacy of ethinyl estradiol as an adjuvant agent in the treatment of premenopausal women with chronic shizophrenia	Inpatients in the active phase of illness, meet the DSM-IV criteria for chronic schizophrenia	Positive and negative symptoms as measured on the PANSS and the (extrapyramidal symptoms rating scale) ESRS	Patients were randomly allocated to receive haloperidol 15 mg/day plus ethinyl estradiol 0.5 mg/day or haloperidol 15 mg/day plus placebo	8 weeks	Positive Symptoms<br>The difference between the two protocols was significant as indicated by the effect of group, the between-subjects factor (Greenhouse –Geisser corrected: F = 6.17, df = 1, P=.019).<br><br>Negative Symptoms<br>The difference between the two protocols was not significant as indicated by the effect of group, the between-subjects factor (Greenhouse –Geisser corrected: F = 0.088, df = 1, P=.76).	Patients who received the ethinyl estradiol showed better clinical outcomes with respect to positive symptoms, general psychopathological symptoms, and PANSS total scores
Bengtsdotter 2018	Sweden	University hospitals	RCT	202	To assess whether women with ongoing or previous mental health disorders or risk use of alcohol at baseline are at higher risk for COC-induced mood symptoms.	Healthy women (18–35 years) with a body mass index between 17 and 30 kg/m2 who accepted to use back-up contraception. Ongoing or previous mental disorders and present use of psychotropic drugs were not reason for exclusion.	Primary outcome- change scores in daily, prospective symptom ratings of 5 mood symptoms on the DSRP scale.<br>The presence of ongoing primary depressive and anxiety disorders as well as eating disorders was established by use of the Swedish 6.0.0 version of the Mini International Neuropsychiatric Interview	COC (1.5 mg estradiol and 2.5 mg nomegestrolacetate) or placebo during three 24/4 treatment cycles	84 days	Women with ongoing or previous mood, anxiety or eating disorders allocated to COC had higher total DRSP D-scores during the intermenstrual phase of the treatment cycle in comparison with corresponding women randomised to placebo, mean difference 1.3 (95% CI 0.3–2.3). In contrast, among women without mental health problems, no difference in total DRSP D-scores between COC- and placebo users was noted. Women with a risk use of alcohol who were randomised to the COC had higher total DRSP D-scores than women randomised to placebo, mean difference 2.1 (CI 95% 1.0–3.2).	
Blasey 2011	US and Europe	40 outpatient clinical research centers across the United States and 5 sites in eastern Europe.	RCT	433	To evaluate the safety and efficacy of mifepristone for the reduction of psychotic symptoms in patients with psychotic depression.	Eligible participants were between 18 and 75 years and were not on antidepressants, antipsychotics, and/or mood stabilizers for at least 7 days before randomization.	Responders were defined as patients with a 50% or greater reduction in their baseline PSS score on days 7 and 56. Patients with less than a 50% reduction from baseline at either day 7 or 56 were defined as nonresponders. Patients requiring rescue treatment with antipsychotic or mood stabilizer medications were also defined as nonresponders.	Patients were randomly assigned 1:1:1:1 to receive either active treatment of mifepristone at 1 of 3 dose levels (300 mg, n =107; 600 mg, n = 107; 1200 mg, n = 109) or placebo (n = 110)	56 days	Mifepristone was well tolerated at all 3 doses. The proportion of responders randomized to mifepristone did not statistically differ from placebo. Patients with trough mifepristone plasma concentrations greater than 1660 ng/mL were significantly more likely to have a rapid and sustained reduction in psychotic symptoms than those who received placebo. The study failed to demonstrate efficacy on its primary end point. However, the replication of a statistically significant linear association between mifepristone plasma concentration and clinical response indicates that mifepristone at sufficient plasma levels may potentially be effective in rapidly and durably reducing the psychotic symptoms of patients with psychotic depression.	.
Chan 1994	United States	A hospital in California	RCT	7	To test whether progesterone or progesterone receptors are important are important mediators pf premenstrual syndrome (PMS) and whether progesterone antagonist RU 486 would alleviate symptoms.	Women with PMS	Symptoms were evaluated using the Calender of Premenstrual Experiences, Beck Depression Inventory, State-Trait Anxiety Inventory, and the Profile of Mood States.	3 months of low-dose RU 486 (5 mg alternate days for 4 doses, beginning 3 days after the urinary LH surge)	8 months	There were no significant differences on any of these measures between placebo and RU 486.	
Comasco 2021	Sweden	Departments of Obstetrics and Gynecology at Uppsala University Hospital, Karolinska University Hospital, Danderyd University Hospital, and Umeå University Hospital	RCT	95	To investigate the usefulness of UPA for treatment of Premenstrual dysphoric disorder	Women were eligible if they were 18–46 years of age,healthy, had regular menstrual cycles, and met criteria for PMDD	The change from baseline in premenstrual DRSP total score (Daily Rating of Severity of Problems)<br>Depressive symptoms, mood swings, and anger/irritability from the DRSP subscale<br>Functional impairment<br>MADRS-S score Montgomery Åsberg Depression Rating Scale<br>EQ-VAS Score EuroQoL visual analogue scale	5mg/day of UPA or placebo during three 28-day treatment cycles	3 months	Previously mentally healthy HC users had an OR of 1.79 for use of antidepressants compared with nonusers, whereas this number was 1.28 for women with previous mental health issues. The highest antidepressant use were uniformly found in strata with previous mental health issues, with highest usage in women aged 24–30 with no immigrant background, low income and HC use (51.4%). The largest difference in antidepressant use between HC users and non-users was found in teenagers, and in adult women of immigrant background with low income. Of the total individual variance in the latent propensity of using antidepressant 9.01% (healthy) and 8.16% (with previous mental health issues) was found at the intersectional stratum level	
Eisenlohr-Moul 2017	United States	A single universitybased medical research center in North Carolina	Doubleblind, randomized, placebo-controlled trial	55	To compare placebo, intermittent dosing of oral contraceptives, and continuous dosing of contraceptives for the treatment of premenstrual dysphoria (PMD)	Women with prospectively confirmed PMD	The Daily Record of Severity of Problems (DRSP)	(1)placebo, (2) intermittently dosed Drospirenone/estradiol 3 mg/20 ug (21/7), and (3) continuous DROS/EE 3 mg/20 ug for 3 cycles.	4 months	Across outcomes, premenstrual symptoms declined significantly in all groups, and these marked declines over time did not significantly differ by treatment group.	
Freeman 2002	United States	Private/Urban	RCT	82	To evaluate the efficacy of DRSP/EE in the treatment of PMDD	The women, ages 18–40, who were otherwise healthy were recruited through advertising and from physician offices. In addition to the PMDD symptom criteria, they were required to have had ovulatory cycles between 25 and 34 days in length for the preceding 3 months, documented by serum progesterone levels; have a negative pregnancy test prior to the first dose of study medication; and use a barrier contraceptive method	The primary outcome measure was the change from baseline in the luteal phase factor 1 (mood) score. Secondary outcome measures were the luteal phase factors 2–4 scores, the total luteal phase COPE score, the 22-item Beck Depression Inventory (BDI), and the Profile of Mood States (POMS)(assessed at baseline and during the follicular and luteal phases of each treatment cycle).	Treatment with drospirenone (DRSP, 3 mg) and ethinyl estradiol(30 mg) (n= 42) or placebo (n= 40)	3 months	Patients treated with DRSP/EE showed a numerically greater change from baseline compared with those treated with placebo on each of the 22 COPE items and each of the 4 symptom factors. Between-group differences in symptom improvement reached statistical significance in factor 3 only (appetite, acne, and food cravings, p 5 0.027). The secondary end points, Beck Depression Inventory (BDI) and Profile of Mood States (PMS), were consistent with the primary end point in that patients treated with the oral contraceptive showed a numerically greater improvement from baseline compared with those treated with placebo.	
Ghafari 2013	Iran	Private	RCT	32	Evaluate the effectiveness of estrogen as an adjuvant agent in the treatment of women with chronic schizophrenia	Childbearing-age women with chronic schizophrenia in an institution for chronic mental illness	Mean PANSS Positive Symptoms	Conjugated estrogens 0.625 mg/day for 4 weeks with previous antipsychotic treatment, versus placebo booster with antipsychotic	4 weeks	Combination of conjugated estrogens with antipsychotic treatment showed significant decrease in positive and negative symptoms.	Estrogen may be an effective adjuvant agent for treating women with chronic schizophrenia.
Gingnell 2013	Sweden	Department of Obstetrics and Gynecology at Uppsala University Hospital	Double-blind randomized, parallel-group clinical trial	34	To investigate if COC use would induce more pronounced mood symptoms than placebo in women with previous history of COC-induced adverse mood. A second aim was to determine if COC use is associated with changes in brain reactivity in regions previously associated with emotion processing.	Healthy women 18-45 years with regular menstrual cycles and subjective reports of mood deterioration during previous COC use	Cyclicity Diagnoser (CD) scale, FMRI scans to detect brain activity, the State-Trait Anxiety Inventory (STAI-S), and the self-rated version of the Montgomery-A˚sberg Depression Rating Scale (MADRS-S).	Women took the COC tablets once daily on the first day of menses and continued treatment for 21 days (oral COC (ethinyl estradiol (EE) 30 mg/0.15 mg levonorgestrel.	2 cycles	During the last week of the treatment cycle (treatment cycle week three under table 2) COC users had higher scores of depressed mood (T = -2.3, p < 0.05), mood swings (T = -3.4, p < 0.01), and fatigue (T = -3.1, p < 0.01) in comparison with placebo users. Irritability and anxious/worried were nonsignificant differences.	

Graham 1992	Canada	A community health center	Double blind placebo controlled trial	59	The major goals were: (1) to assess the efficacy of a triphasic O.C. as a treatment for moderate to severe premenstrual symptoms; (2) to determine whether differential response of women with PMS to an O.C. might be associated with a specific symptom profile and; (3) to assess the effect of blocking ovulation on the timing and severity of premenstrual symptoms in an effort to elucidate the mechanisms underlying PMS.	Women with PMS who were seeking treatment for self-reported moderate to severe physical and psychological premenstrual symptoms	Shortened 15 item version of the daily ratings form was used to monitor changes daily, 9 visual analog scales noted intensity of mood and physical symptoms, and The Post-treatment Questionnaire	A fixed dose of ethinyl estradiol 0.035 mg from days 1-21 and norethindrone, 0.5 mg during days 1-7, 1 mg during days 8-16, and 0.5 mg during days 17-21 for 4 cycles.	5 cycles	There was a decrease in menstrual phase scores for four variables (‘mood swings’, ‘more sleep’, ‘unhappy’ and ‘tense’) in the second treatment cycle compared to the first month on treatment, irrespective of treatment group. However, scores in postmenstrual and intermenstrual phases did not change across the trial. There was a significant reduction of premenstrual breast pain among OC group compared to placebo. Same pattern seen in the reduction of sexual interest.	
Graham 1993	Canada	Teaching Hospital	RCT	45	Association between Mood and Sexuality for oral contraceptive users	Women 18-35 years old, with no current or chronic medical or gynelogical disorders, not currently receiving treatment from mental health professionals, not taking psychotropic or hormonal medications, having regular menstrual cycles, not currently using OCs, not pregnant or planning a pregnancy, show prospective confirmation of moderate to severe premenstrual changes	Changes in self-reported moderate to severe physical and psychological premenstrual symptoms	Triphasic oral contraceptive from days 1-21 compared to a placebo group containing lactose and cornstarch for 3 cycles	3 cycles	No significant data to extract.	No significant correlation between depression and sexual interest for users or placebo group
Halbreich 2012	US and Canada	This multicenter study was conducted at 71 sites in the United States and 14 sites in Canada.	Randomized, double-blind, placebo-controlled study	367	To investigate continuous daily levonorgestrel 90 mcg/ethinyl estradiol 20 mcg (LNG/EE) on premenstrual dysphoric disorder (PMDD)	Healthy women aged 18 to 49 years who met DSM-IV-TR criteria for PMDD over the preceding year	DRSP score during the late luteal phase and worst 5 days	Continuous daily oral LNG 90 mcg/EE 20 mcg tablets for 112 days (4 consecutive 28-day pill packs)	112 days	Mean DRSP change from baseline to late luteal phase was significantly greater with LNG/EE than placebo at the late luteal phase of the first estimated cycle (−30.52±1.73 [SE] vs. −22.47±1.77; p < .001) and the worst 5 days during the last on-therapy estimated cycle (−26.77±1.83 vs. −20.89±1.82; p=.016).	
Joffe 2007	United States	Participant recruitment took place at community and psychiatry outpatient clinics between 2004-2005	Randomized, double-blind, placebo-controlled trial	25	To determine whether stabilization of estradiol and progeserone levels with an OCP treats depression symptoms that break through premenstrually in women with depression that is otherwise well controlled by antidepressants.	Women aged 18-45 with regular 26-35 day menstrual cycles predictable within 7 days, no hormonal contraceptive use within the past 6 months, onset of depressive disorder at least 3 months prior to study participation, use of current antidepressant for at least 3 months, depressive disorder in full remission for at least 2 months with exception of premenstrual week, and willingness to use barrier contraceptive methods.	The primary outcome was the percent change in the mean premenstrual Montgomary-Asberg Depression Rating Scale (MADRS) score from baseline (Average of last 5 days prior to mense during the run-in period) to the second treatment month (obtained on days 25-28 of the OCP cycle and reflecting the week prior to the withdrawal bleed).	21 days of EE 30 µg/day plus DRSP 3 mg/day with double-blinded assignment to daily EE 30 µg (EE/DRSP+EE) or placebo (EE/DRSP+placebo) during the final 7 days of each 28-day cycle.	3 months	Prementstrual MADRS (p = 0.0019) and Daily Rating of Severity of Problems scores (p=0.0001) improved significantly in both groups and did not differ between the treatment and placebo groups.	Authors concluded that the addition of EE/DRSP (+/- EE) to antidepressants may treat premenstrual breakthrough of depression.
Lascurain 2020	Spain	The patients were recruited over a 3-year period between 2006 and 2011.	Three-arm, double-blind, randomised-controlled trial	180	To investigate the effect of oestradiol treatment on comorbid depressive symptoms in women with schizophrenia.	Women aged 14-45 with schizophrenia and ongoing symptoms of psychosis Positive and Negative Syndrome Scale (PANSS) score > 60 despite a stable dose of antipsychotic medication	The primary outcome was the Positive and Negative Syndrome Scale (PANSS) score, as well as the Montgomery Asberg Depression Scale (MADRS) score.	Participants randomized to the treatment group either received 100 or 200 µg oestradiol (administered as transdermal patches). Placebo patches were adhesive and identical in appearance but had no active substance. Patches were changed every 3.5 days.	8 weeks	The MADRS depression scores comparing oestrogen 200 mcg to placebo fluctuated between borderline significance (p = 0.05) at day 7, to non-significant at day 14 (p = 0.07) to significant at day 28 and back to borderline significance at day 56. There were no significant differences found comparing oestrogen 100 mcg with oestrogen 200 mcg or oestrogen 100 mcg with placebo at other time points.	Authors concluded adjunctive oestradiol treatment for depression may be a promising treatment for women with comorbid depression and schizophrenia.
Lundin 2017	Sweden	Uppsala University Hospital, the Karolinska University Hospital, Södersjukhuset, Linköping Uni-versity Hospital, Örebro University Hospital, Umeå UniversityHospital, and Närhälsan Maternity Health Care Center in Frölunda, Gothenburg, between September 7, 2013 and September 29, 2015.	Randomized, double-blinded, placebo-controlled study	202	To estimate the severity of adverse mood in COC users that would be as representative of general users as possible.	Women aged 18-35 with a body mass index below 30 kg/m2 who accepted use of back-up contraception during the study period.	The primary outcome was the Daily Record ofSeverity of Problems (DRSP), which was filled out daily during one baseline cycle and the final treatmentcycle.	Participants were randomized to 1.5 mg estradiol and 2.5 mg nonmegestrolacetate or placebo during three 24/4 treatent cycles (24 days of treatment, followed by 4 pill-free days).	84 days	Increases in mean anxiety (0.22; 95% CI:0.07–0.37, p = 0.003), irritability (0.23; 95% CI: 0.07–0.38, p = 0.012), and mood swings scores (0.15; 95%CI: 0.00–0.31, p = 0.047) in the COC group compared to the placebo group were observed during the intermenstrual phase. However, a significant premenstrual improvement in depression (−0.33; 95% CI: −0.62 to −0.05, p = 0.049) was observed in the COC group compared to the placebo group.	Authors concluded COC use is associated with small but statistically significant mood side effects in the inter-menstrual phase. However, positive mood effects are noted in the premenstrual phase and the proportion of women with clinically relevant mood worsening did not differ between treatment groups.
Marr 2011	United States	Multicenter	RCT	449	Cycle by cycle changes of symptoms of PMDD with combined oral contraceptive	Women aged 18-40 years with an objectively confirmed diagnosis of PMDD	Negative emotions associated with diagnosed PMDD	Treatment with EE 20 mcg/drospirenone 3 mg 24/4 or placebo	3 cycles	Mean (SD); Negative emotions baseline for the intervention group was 26.6 (4.50) compared to a mean of 20.70 (4.42). Negative emotions were measured at cycles 1, 2, and 3. At cycle 1, the intervention group had a mean of 10.72 (5.70) and the control group had a mean of 13.32 (6.68). At cycle 2 the intervention group had a mean of 9.86 (5.44) and the control group mean was 11.93 (6.65). At cycle 3, the intervention group had a mean value of 9.36 (5.44) and the control group had a mean value of 12.10 (6.88)	EE 20 mcg/drospirenone 3 mg 24/4 significantly improves three commonly recognized PMDD symptom groups (negative emotions, food cravings and water retention-related symptoms) compared with placebo in all three cycles of treatment
Pearlstein 2005	United States	24 centers in the United States	Multicenter, double-blind, placebo-controlled crossover study	64	To evaluate the efficacy of a new oral contraceptive (OC) formulation containing drospirenone 3 mg and ethinyl estradiol (EE) 20 ug in treating symptoms of premenstrual dysphoric disorder (PMDD)	Women with moderate to severe premenstrual symptoms	The primary outcome measure consisted of the 21 symptom items of the DRSP, which are rated on a six-point scale from 1 (not at all) to 6 (extreme). Secondary outcome measures included the three functional impairment items of the DRSP and the Premenstrual Tension Scales observer-rated (PMTS-O) and self-rated (PMTS-SR) scales.	The OC formulation (Drospirenone 3 mg and ethinyl estradiol 20 ug) or placebo was administered for 24 days in a 28-day cycle (24/4) for three cycles and then after a washout period of one treatment-free cycle switched to the alternate treatment.	7 cycles	Women who began with the D/EE treatment initially, reported a 51% improvement in mood compared to 31% in the placebo group. After cross over, mood improved 34% for the D/EE group, and women who were switched to the placebo saw a 17% worsening of their mood.	This was a cross over study design – so people were randomized to either begin with placebo or intervention group, then after a 3 week washout period, were switched to the other intervention.
Peters 2017	United States	The Center for Women's Mental Health at Massachusetts General Hospital	Randomised Controlled Trial	25	To confirm that stabilizing levels of estradiol and progesterone with an adjunctive regimen of DRSP/EE would be more effective than placebo in treating premenstrual breakthrough of depressive symptoms in women whose depression is otherwise well controlled by antidepressants.	Women 18 to 45 years old with regular menstrual cycles who were taking anti depressants for the treatment of unipolar depression	Change in median premenstrual MADRS from baseline to second treatment month. Secondary outcome is the change in median premenstrual DRSP from baseline to second month of treatment	2 months of either the OCP DRSP/EE (each pill pack containing 24 days of 0.02 mg ethinyl estradiol [EE] with 3.0 mg drospirenone [DRSP] per day followed by 4 days of placebo) or an identical placebo.	3 months	No statistically significant between-group differences in the change in premenstrual QOLI, SDS, and CGI scores with treatment (all P>0.14). Premenstrual MADRS scores declined by a median of 43.6% and 38.9% (P = 0.59), and premenstrual DRSP scores declined by a median of 23.5% and 20.9% (P = 0.62) in the OCP and placebo groups, respectively.	

Petersen 2021	United States	Participants recruited via Internet advertisements	Double-blind, placebo-controlled, randomized crossover study	26	To determine whether there is an effect of OCPs on MRI measures of prefrontal cortical brain structure that may influence regulation of mood.	Healthy women who had reported previous mood deterioration while using OCs	MRI measures of prefrontal cortical thickness and self-report on the Daily Record of Severity of Problems.	21 OC pills (0.30 µg ethinyl estradiol/0.15 mg levonorgestrel) to be taken once daily, starting on the first day of menses	3 cycles	18–21 days of OCP use indeed reduced prefrontal cortical thickness, with the most pronounced effect in the right inferior frontal gyrus; OCPs also produced negative mood symptoms as self-reported on the BDI and DRSP.	Oral contraceptives significantly increased total score of self reported symptoms on the DRSP.
Scheuringer 2020	Sweden	University Hospital	RCT	69	Determine if combined oral contraceptives impact emotions and mood and predict factors which may influence depressive symptoms	Somatically healthy women aged 18-35 years, BMI <30.0 kg/m2, willing to use back-up contraceptives during the study period	MADRS for self rated depression score via psychological and health questionnaires; includes depression, anxiety, neurotic personality, PMDD, neuropsychiatric interview (ogoing or previous mood changes)	Participants treated with COC containing 1.5 mg estradiol and 2.5 mg nonmegestrolacetate or placebo for 3 consecutive 24/4 treatment cycles.	3 months	Strongest predictor for depressive symptoms were trait anxiety at baseline (B = 0.35, *B* = 0.59, SE = 0.07). Also, regardless of treatment group, prior negative mood experience of oral contraceptive use also affected depressed mood at the end of the trial (B = 2.30, *B = 0.22,* SE = 1.06)	None of the treatment interactions were significant.
Weiser 2019	The Republic of Moldova	The psychiatric hospital in Moldova	Randomized, placebo-controlled trial	100	To independently replicate the results of the effect of estradiol on schizophrenia in women of childbearing age.	Women of childbearing age (19 to 46 years) with schizophrenia or schizoaffective disorder	The primary outcome was the positive subscale of the Positive and Negative Syndrome Scale (PANSS; lower scores indicated fewer symptoms and higher scores indicated more symptoms).	A 200-µg estradiol patch or placebo patch changed twice a week added to their antipsychotic treatment for 8 weeks	8 weeks	The estradiol group showed statistically significant improvement in the primary outcome measure, the difference in PANSS positive subscale scores (–0.94; 95% CI, –1.64 to –0.24; P = .008; effect size, 0.38), and in the secondary outcome measures of PANSS total score (difference, –4.10; 95% CI, –6.73 to –1.47; P = .002; effect size, 0.45) as well as the PANSS subscales for negative symptoms and general psychopathology compared with the placebo group. There were also significantly greater improvements with the estradiol patch relative to placebo in the CGISeverity score and MADRS score.	Clinical Global Impression Scale: 3 item scale used to assess global illness severity, overall improvement from the start of treatment and therapeutic response.<br>Brief Assessment of Cognition in Schizophrenia: assesses the aspects of cognition found to be most impaired and most strongly correlated with outcome in patients with schizophrenia.<br>Montgomery Depression scale: 0 to 6 indicates that the patient is in the normal range (no depression), a score ranging from 7 to 19 indicates "mild depression," 20 to 34 indicates "moderate depression," a score of 35 and greater indicates "severe depression"<br>PANSS, Positive and Negative Syndrome Scale : s a medical scale used for measuring symptom severity of patients with schizophrenia.
Yonkers 2005	United States	64 centers in the United States	Double-blind, randomized clinical trial	450	To compare the efficacy of a new low-dose oral contraceptive pill (OCP) formulation with placebo in reducing symptoms of premenstrual dysphoric disorder.	Women with symptoms of premenstrual dysphoric disorder	The difference between the average luteal phase Daily Record of Severity of Problems total scores from the 2 qualification cycles and the average luteal phase Daily Record of Severity of Problems total scores from the 3 treatment cycles.	An OCP formulation containing drospirenone 3 mg and ethinyl estradiol 20 ug. Hormones were administered for 24 days, followed by 4 days of inactive pills (24/4) for 3 cycles.	5 cycles	The estimated adjusted mean change from baseline over 3 cycles in the total Daily Record of Severity of Problems symptom score was –37.49 for the drospirenone/ethinyl group and – 29.99 for the placebo group, for an adjusted mean difference of –7.50 (95% confidence interval [CI] –11.2 to –3.8; P < .001 by rank ANCOVA).	
Zethraeus 2017	Sweden	Single Center University Hospital	RCT	340	Influence of combined oral contraceptives on general well being in health women	Women willing to start using OCs; 18-35 years old, BMI 19-30, a regular menstrual cycle (25-33 days), using non-hormonal contraceptives at the start of the study, fluent in Swedish	Primary outcome is well-being using the normalized global score of the PGWBI; Secondary outcome is normalized scores on each PGWBI dimension: anxiety, depression, mood, positive well-being, self-control, general health, vitality<br>**PGWBI consists of 22 items - each item has 6 response choices and the value of each item ranges from 05. Also has 6 dimensions, global score varies from 0 (poor quality of life) to 110 (good quality of life)	Combined monophasic pill (Neovletta) containing 150 mg levonorgestrel and 30 mg ethinylestradiol during a period of 3 months (n = 169) compared to placebo group (n = 171)	3 months	No data available except baseline characteristics.	Study is limited by short duration. Results do not provide information on mean

Table S2: Characteristics of Observational Studies

Study (year)	Country	Study setting (public /private or rural/urban)	Study design	Sample size	Study aim	Population	Outcome (s) of interest	Exposure (observational studies)	Period of observation (weeks, months, years)	Findings (effect size and 95%CI)	Note
Abraham 2003	Australia	Family doctors	Cohort study	119	To examine the cyclicity and group differences in daily menstrual cycle mood and physical experiences of three groups of 'healthy women' using monophasic, triphasic OC or non-hormonal contraception, and to investigate the interaction of follicular depression scores with premenstrual and menstrual exacerbation of experience ratings.	Women English speaking, aged 18-41 years and had regular menstrual cycles (23-35 days)	Menstrual cycle experiences were recorded prospectively using a daily diary, containing 15 physical experiences scored from absent (0) to very severe (5), 6 mood experiences scored from absent (0) to present all the time (9), and 2 general experiences scored from absent (0) to very severe (5)	Monophasic OC (30 pg ethinylestradiol and 150 pg levonorgestrel, monophasic group), triphasic OC (30/40/30 pg ethinylestradiol and 50/75/125 pg levonorgestrel, triphasic group) or no hormonal contraception (and no intrauterine device, non-OC user group)	2 complete menstrual cycles	There were no significant differences between the three groups in cyclic changes for any physical rating, but there were for tiredness or fatigue (non-OC users reported experiencing tiredness or fatigue more frequentiy than the OC users) and sadness or depression (non-OC users experienced sadness or depression less frequently than OC users during the early part of the cycle, followed by a sharp rise from early premenstrual to the menstrual phase). There were no significant cyclic differences in ratings between the monophasic and triphasic groups.	
Beral 1999	Britain	General Practices	Cohort study	46,000	Mortality associated with oral contraceptive use	Women recruited from general practices throughout the UK; users or non users of oral contraceptives; most are white, all are married	Mortality; Deaths related to cancers of the breast, cervix, uterus, and ovary. Deaths related to suicide	Oral contraceptives	Mean follow up time was 25 years.	Relative risk of mortality due to breast cancer 1.1 (95% CI = 0.8-1.4); Relative risk of mortality due to cervical cancer 1.7 (95% CI 0.9-3.2); RR due to uterine cancer 0.3 (95% CI 0.1 to 1.4); RR due to ovarian cancer 0.6 (95% CI 0.3-1.0); RR due to suicide 1.5 (95% CI 0.8-2.7)	
Castilho 2015	United states	Primary and subspecialty health care HIV clinic in the United states	Retrospective cohort study	392	To describe the isk of psychiatric and other noninfectious complications of hormonal contraception use in HIV-infected women.	HIV-1 infected women	Incident psychiatric diseases and NCDs (cardiovascular disease, renal disease, hepatic diseases, metabolic diseases, and non-AIDS-defining malignancies)	Hormonal contraception	A median follow-up of 42 months in hormonal contraception users and 15 months in nonusers	Compared with no hormonal contraception exposure, the incidence of psychiatric events was 2-fold higher during person-time with DMPA exposure (p = 0.02) but was not statistically different during exposure time with combined hormonal contraception.	In this study, mood disorders include depression and bipolar affective disorder.
Charlton 2014	United States	Mixed	Prospective cohort study	121,577	Determine the impact of oral conctraceptives on all casuses and cause specific mortality	Women recruited through the Nurse's Health Study. Married female registered nurses aged 30-55	All cause mortality, deaths organized into six major categories defined by ICD-8 codes. Two major categories of interest were cancer and violent or accidental deaths (suicide)	Oral Contraceptives (Ever Use, dosage not specified)	Women were followed for 36 years; Lifetime oral contraceptives use was recorded biennially from 1976 to 1982	Violent or accidental deaths were more common among ever users of oral contraceptives (HR 1.20, 95% CI 1.04, 1.37). Longer durations of use of oral contraceptives were associated with premature mortality due to breast cancer (P<0.0001) and decreased mortality rates of ovarian cancer (P=0.002). More time since last use of oral contraceptives was associated with violent/accidental deaths (P=0.005)	No association seen between ever use of oral contraceptives and all cause mortality.
Colditz 1994	United States	Nurses' Health Study	Prospective cohort study	166,755	To examine prospectively the risk for mortality among women who had ever used oral contraceptives compared with those who had never used oral contraceptives.	Female registered nurses aged 30 to 55 years in 1976	All deaths including a category for cancer (breast, endometrium, and ovary)	Oral contraceptive	1.3 million person-years	Mortality caused by breast cancer was not elevated among ever-users of oral contraceptives compared with never-users (relative risk, 1.07; CI, 0.86 to 1.34), and endometrial uterine cancer mortality showed an apparent but nonsignificant reduction (relative risk, 0.33; CI, 0.10 to 1.11).	
deWit 2020	Netherlands	The third to sixth wave of the prospective cohort study Tracking Adolescents' Individual lives Survey (TRAILS)	Prospective cohort Study	1,010	To investigate the association between oral contraceptive use and depressive symptoms and to examine whether this association is affected by age and which specific symptoms are associated with oral contraceptive use.	Adolescent girls in the Netherlands	Depressive symptoms assessed by the DSM-IV-oriented affective problems scale of the Youth (aged 16 years) and Adult Self-Report (aged 19, 22, and 25 years).	Oral contraceptives	10 years	Overall, however, OC use showed no association with depressive symptoms when all age groups were combined, but 16-year-old females reported higher depressive symptom scores. All users combined (mean [SD] ages 16.3 [0.7] to 25.6 [0.6] didn't show higher depressive symptom scores compared with nonusers. Adolescent users mean [SD] age 16.5 [0.7] reported higher depressive symptom scores compared to their nonusing counterparts (mean age 16.1 [0.6] years, which persisted after adjusting for age, SES, and ethnicity. Adolescent users reported more crying g (odds ratio, 1.89; 95% CI, 1.38-2.58; P < .001), hypersomnia (odds ratio, 1.68; 95% CI, 1.14-2.48; P = .006), and more eating problems (odds ratio, 1.54; 95% CI, 1.13-2.10; P = .009) than nonusers.	Study also reported on the correlation of SES and ethnicity with depressive symptoms scores.
Ditch 2019	United States	Private	Cohort study	Control Group 1 n = 269,078, Control Group 2 (n = 94,700), Hormonal Contraceptiv e Users (n = 3,615)	Association of hormonal contraception with depression and antidepressant use	Women aged 12-34 years, enrolled in the United States Military Healthcare System (MHS)	Diagnosis of depression, initiation of antidepressants	Pill, patch, ring, IUD, implant, injectable	Secondary analysis of insurance records; 1 year of follow-up	Compared to control group 1, higher depression rates were seen among women using Norgestimate, Levonorgestrel, Etonogestrel, or Norelgestromin. Compared to control group 2, levonogestrel and Norelgestromin use was associated with higher rates of depression (1.42 (1.05-1.92), p = 0.024 and 1.93 (1.04-3.60), p=0.037). Use of Norethindrone containing contraception was protective (0.21 (0.05-0.85), p=0.028). Compared to control group 1, use of Norgestimate, Levonorgestrel, or Nogestrel was associated with a higher hazard of SSRI use.	There an is an association between initating hormonal contraception and subsequent diagnoses of depression or SSRI use. Associations were diminished when analyses were restricted to women who accessed care.
Ditch 2020	United States	Private/Military	Retrospective cohort study	272,693	Association between contraception initation and diagnosis of depression	Women aged 12-34 who initiated hormonal contraception enrolled in the United States Military Healthcare System	Diagnosis of depression and dispensing of antidepressant medications	Hormonal contraceptives.	Women were followed for 12 months.	Among women who accessed care during the study period, progestins (levonorgestrel (HR = 1.43) and norelgestromnin HR = 1.93)) were associated with increased rate of depression diagnosis but not antidepressant use. Norethindrone (HR = 0.21) was associated with decreased rate of diagnosis.	Women who initiated hormonal contraception experienced a higher risk of depression diagnosis and antidepressant use than all enrolled women, but not compared to women who accessed care.
Duke 2007	Australia	Population Based - Rural	Longitudinal cohort study	Users from survey in year 2000 (n= 9688), Users from	Association of oral contraceptive use and depressive symptoms	Australian women in the young age group cohort ("young" 18-23 years)	Score on Center for Epidemiologic Studies Depression scale (CESD-10)	Oral contraceptive pills	20 years of follow-up	Non-users of oral contraception were used as the reference category. The odds of a non-user experiencing depressive symptoms were 1.43 (95% CI = 1.28-1.58) times that of an OCP user. After adjustment for confounders, women who used OCP for reasons other than contraception were 1.32 (95% CI = 1.07-1.62) times as likely to report	Nonsignificant results when adjusted for factors known to be associated with depression.
Edwards 2020	Sweden	Swedish national registry	Cohort study	216,702	To assess whether OC use among young women aged 15–22 is associated with risk of suicidal behavior	All females in Sweden who turned 15 in august 2006 or later	Age at first suicide event	Oral contraceptive	Around 7 years	Use of combination or progestin-only oral contraceptives was positively associated with suicidal behavior, with hazard ratios (HRs) of 1.73–2.78 after 1 month of use, and 1.25–1.82 after 1 year of use	
Hamstra 2017	Netherlands	At the campus of Leiden University	Prospective longitudinal study	92	To assess aspects associated with reproductive depression such as mood, interpersonal sensitivity, affect lability and depressive cognitions in MR-genotyped OC-users and naturally cycling (NC) women	Healthy, PMS-free, pre-menopausal MR (mineralocorticoid receptor) -genotyped women	Interpersonal sensitivity, affect lability, cognitive reactivity, and positive and negative affect states	Monophasic pills with as compounds ethinylestradiol (EE; 0.03)/levonorgestrel (LNG; 0.15) for more than three months and applied a pillfree week.	2 months	OC-users did not differ significantly from NC women in positive and negative affect at the time of assessment, personality characteristics (e.g. neuroticism) or mental and physical health.	

Hannaford 2010	United Kingdom	1400 general practitioners (GPs) throughout the United Kingdom between May 1968 and July 1969	Prospective cohort Study	Around 46,000	To see if the mortality risk among women who have used oral contraceptives differs from that of never users	Women who were using the pill and a similar number who had never used this method of contraception	Directly standardised adjusted relative risks between never and ever users for all cause and cause specific mortality	Oral contraceptive	Up to 39 years	Compared with never users, ever users of oral contraception had a significantly lower rate of death from large bowel/rectum (RR 0.62, 95% CI 0.46 - 0.83), uterine body (RR 0.43, 95% CI 0.21 - 0.88), and ovarian cancer (RR 0.53, 95% CI 0.38 - 0.72).	
Lundin 2021	Sweden	Cohort identified from from six Swedish national population-based registers.	Register-based cohort study	739,585	To investigate whether users of hormonal contraceptives (HCs) are at increased risk of depression compared with nonusers.	Women aged 15–25 years between 2010 and 2017 with no prior antidepressant treatment, psychiatric diagnose or contraindication for hormonal contraceptives.	The primary outcome was depression, measured through the redemption of a prescription of antidepressant treatment, or a first depression diagnosis in the SPDR and the National Patient Register.	Women who were treated with any HC between 1 January 2010 and 31 December 31 2017.	All women were followed upon entry in the study (1 January 2010, or on their 15th birthday) until event, emigration, death or the end of the follow up in (31 December 2017, or on their 26th birthday).	Compared with non-users, women on combined oral contraceptives (COCs) and oral progestogen-only products had lower or no increased risk of depression, relative risk (RR 0.89, 95%CI 0.87–0.91) and (1.03, 95%CI 0.99–1.06) after adjustments, respectively.	Authors concluded that there was no observable association between use of COCs (the most popular HC in first-time users) and depression. Non-oral products, however, were associated with increased risks.
Oinonen 2001	Canada	A university	Prospective cohort study	96	The relationship between affect and duration of oral contraceptive (OC) use was investigated.	Female university students	Initial General Information Questionnaire, Daily Rating Questionnaire (DRQ), and Final General Information Questionnaire	Oral contraceptive	35 days	No differences in positive or negative affect were found between the three groups of women over the entire menstrual cycle or at any specific phase of the cycle. The first-time users indicated experiencing significantly more negative effects than the long-time user group.	
Skovlund 2016	Denmark	A nationwide prospective cohort study combined data from the National Prescription Register and the Psychiatric Central Research Register	Prospective cohort study	1,061,997	To investigate whether the use of hormonal contraception is positively associated with subsequent use of antidepressants and a diagnosis of depression at a psychiatric hospital.	All women and adolescents aged 15 to 34 years who were living in Denmark	Adjusted incidence rate ratios (RRs) were calculated for first use of an antidepressant and first diagnosis of depression at a psychiatric hospital.	Hormonal contraception	A mean [SD] follow-up of 6.4 [0.004] years	Compared with nonusers, users of combined oral contraceptives had an RR of first use of an antidepressant of 1.23 (95% CI, 1.22-1.25). Users of progestogen-only pills had an RR for first use of an antidepressant of 1.34 (95% CI, 1.27-1.40); users of a patch (norgestrolmin), 2.0 (95% CI, 1.76-2.18); users of a vaginal ring (etonogestrel), 1.6 (95% CI, 1.55-1.69); and users of a levonorgestrel intrauterine system, 1.4 (95% CI, 1.31-1.42). For depression diagnoses, similar or slightly lower estimates were found.	
Skovlund 2018	Denmark	Population based - Urban	National Cohort study	475,802	Assess the influence of hormonal contraceptive use on suicides	Women in Denmark who turned 15 during the study period (1996 to 2013), with no prior use of hormonal contraceptive use	Suicide attempt or suicide	Hormonal Contraceptives	Women were followed for an average of 8.3 years (3.9 million person-years)	Compared to never-users, users of hormonal contraception 15-33 years of age had a relative risk of 1.97 (95% CI = 1.85-2.10) for a first suicide attempt and 3.08 (95% CI = 1.34-7.08) for suicide. Former users were found to have a relative risk of 3.40 (95% CI=3.11 - 3.71) for a first suicide attempt, and 4.82 (95% CI=1.93-12.1) for suicide.	Hormonal contraceptives were positively associated with a first suicide attempt, compared to never use. Adolescent users demonstrated highest risk.
Yonkers 2017	United States	Multicenter	Cohort study	The Screened Cohort: 490 women; the Randomized Cohort: 252 women	To determine if premenstrual symptoms differ between women who use cyclic hormonal contraception and those who do not	Women aged 18-48 years; had menstrual cycles of 21-35 days; could speak and write in English	Daily Rating of Severity of Problems (DRSP) to catalog daily symptoms of premenstrual dysmorphic disorder (PMDD).	Cyclic oral contraceptives	Women were followed from symptom onset until the first few days of menses for 6 menstrual cycles.	Outcomes measured using least square mean. Screened cohort: magnitude of change was greater for the non-CHC group than the CHC group, but significant differences for individual symptoms occurred only for depressed, hopeless, or guilty (p = 0.03); anger or irritability (p = 0.002); diminished interest (p = 0.001); difficulty concentrating (p = 0.03), feeling overwhelmed (p = 0.05), and physical symptoms (p = 0.04). In the Randomized Cohort, there were no significant differences in the magnitude of change between CHC groups for any individual symptoms.	
Young 2007	United States	18 primary and 23 pyschiatric settings across the US-Multicite	Observational study	1238	To determine whether estrogen–progestin combination or progestin-only contraceptives are associated with depression severity, function and quality of life, or general medical or psychiatric comorbidity in women with MDD.	Outpatient women less than 40 years with non-pyschotic MDD	Primary outcome: changes associated with the menstrual cycle such as mood, depression, anxiety, irritability, mood variation, panic, PTSD, # of depressive episodes, hamiliton rating scale for depression, 30 item inventory of depressive symptoms, 16 item quick inventory of depressive symptomatology, 12 item short form health survey, quality of life and satisfaction questionnaire.	Estrogen–progestin combination or progestin-only contraceptives	A mean follow-up of 14.64 months in COC users, 18.54 months in POP users, and 19.74 in non-users	Overall, compared to the nonhormone group, women on combination hormone contraception showed less severity of depression, better physical functioning on the SF-12 and fewer comorbid anxiety disorders based on the PDSQ, though after adjusting for confounding, only OCD was significant.	MDE: Major Depressive Episode, CIRS: Cumulative Illness Rating Scale, HRSD17: 17-item Hamilton Rating Scale for Depression, IDS-C30: 30-item Inventory of Depressive Symptomatology, QIDS-SR16: 16-item Quick Inventory of Depressive Symptomatology, SF-12: 12-item Short Form Health Survey, WSAS: Work and Social Adjustment Scale, Q-LES-Q: Quality of Life Enjoyment and Satisfaction Questionnaire.
Zettermark 2018	Sweden	Population Based	Cohort study	815,662	Association between hormonal contraceptives and adverse psychological health outcomes	All women aged 12-30 years on December 31, 2010, residing in Sweden for at least 4 years, with no previous psychiatric morbidity	Use of psychotropic drugs	Combined oral contraceptives, progestin only pills, IUD/INJ/implant, patch/ring	1 year of follow up	Overall association between hormonal contraceptives and psychotropic drug use (adjusted OR 1.34, 95% CI 1.30-1.37). In age stratified analysis, association was strongest among adolescent girls (adjusted OR 3.46, 95% CI 3.04-4.94 for ages 12-14 years). Association was not found for adult women.	Hormonal contraception is most associated with psychotropic drug use among adolescents, indicating a potential adverse effect of HC in this age group.
Zettermark 2021	Sweden	Population Based	Cohort study	915,954	Association between hormonal contraceptive use and antidepressant use	The entire Swedish population of women aged 12-30	Antidepressant use	Hormonal Contraceptives	1 year of follow up.	Previously mentally healthy HC users had an OR of 1.79 for use of antidepressants compared with nonusers, whereas this number was 1.28 for women with previous mental health issues. The highest antidepressant use were uniformly found in strata with previous mental health issues, with highest usage in women aged 24–30 with no immigrant background, low income and HC use (51.4%). The largest difference in antidepressant use between HC users and non-users was found in teenagers, and in adult women of immigrant background with low income. Of the total individual variance in the latent propensity of using antidepressant 9.01% (healthy) and 8.16% (with previous mental health issues) was found at the intersectional stratum level	

Table S3: Setting of the included studies

Region	Number of studies	Studies (name of author, year of publication)
Africa	0	
Asia	0	
Americas	20	Castilho 2015; Chan 1994; Charlton 2014; Colditz 1994; Ditch 2019; Ditch 2020; Eisenlohr-Moul 2017; Freeman 2002; Graham 1992; Graham 1993; Halbreich 2012; Joffe 2007; Marr 2011; Oinonen 2001; Pearlstein 2005; Peters 2017; Petersen 2021; Yonkers 2005; Yonkers 2017; Young 2007
Eurasia	19	Bengtsdotter 2018; Beral 1999; Comasco 2021; deWit 2020; Edwards 2020; Gingnell 2013; Hamstra 2017; Hannaford 2010; Lascurain 2020; Lundin 2017; Lundin 2021; Scheuringer 2020; Skovlund 2016; Skovlund 2018; Weiser 2019; Zethraeus 2017; Zettermark 2018; Zettermark 2021
Middle East	2	Akhondzadeh 2003; Ghafari 2013
Oceania	2	Abraham 2003; Duke 2007
Combined	1	Blasey 2011

Table S4: Quality Assessment of the Observational Studies

Author, year	Reporting	External validity	Internal validity		Total score
			Bias	Confounding	
Abraham 2003	5	1	5	2	13
Beral 1999	4	1	4	3	12
Castilho 2015	6	1	4	2	13
Charlton 2014	6	2	5	3	16
Colditz 1994	6	2	4	3	15
deWit 2000	6	2	5	3	16
Ditch 2019	3	0	3	3	9
Ditch 2020	5	1	5	3	14
Duke 2007	4	0	3	3	10
Edwards 2020	5	2	5	3	15
Hamstra 2017	4	1	4	2	11
Hannaford 2010	6	1	5	3	15
Lundin 2021	6	1	4	3	14
Oinonen 2001	5	1	4	3	13
Skovlund 2016	6	2	5	3	16
Skovlund 2018	5	1	5	3	14
Yonkers 2017	6	1	4	3	14
Young 2007	6	2	3	3	14
Zettermark 2018	5	2	4	3	14
Zettermark 2021	6	2	4	3	15
