# Supplementary material for: Assessing the impact of contraceptive use on mental health among women of reproductive age – a systematic review
Source: BMC Pregnancy Childbirth. 2024 May 30;24:396. doi: 10.1186/s12884-024-06587-9 (PMC11137968; doi:10.1186/s12884-024-06587-9)
Supplement: Supplementary file 3 — Supplementary Material 3 [file 12884_2024_6587_MOESM3_ESM.docx]

| **Table S5. Summary of findings for three comparative analysis of depression.** | | | | | | |
| --- | --- | --- | --- | --- | --- | --- |
| **Oral contraceptives compared to placebo in Mental Health** | | | | | | |
| **Patient or population:** Mental Health  **Setting:**  **Intervention:** Oral contraceptives  **Comparison:** placebo | | | | | | |
| Outcomes | **Anticipated absolute effects^*^** (95% CI) | | Relative effect (95% CI) | № of participants (studies) | Certainty of the evidence (GRADE) | Comments |
|  | **Risk with placebo** | **Risk with Oral contraceptives** |  |  |  |  |
| Montgomery Åsberg Depression Rating Scale (MADRS) | - | SMD **0.09 lower** (0.34 lower to 0.15 higher) | - | 279 (5 RCTs) | ⨁⨁⨁⨁ High |  |
| Depression without mental disorders [Continious] | - | SMD **0.18 higher** (0.02 higher to 0.34 higher) | - | 591 (3 RCTs) | ⨁⨁⨁◯ Moderate^a^ |  |
| Depression with mental disorders [Continious] | - | SMD **0.15 lower** (0.3 lower to 0 ) | - | 742 (9 RCTs) | ⨁⨁⨁◯ Moderate^a^ |  |
| ***The risk in the intervention group** (and its 95% confidence interval) is based on the assumed risk in the comparison group and the **relative effect** of the intervention (and its 95% CI).  Moderate^a^ we reduced quality due to high heterognetiy.   **CI:** confidence interval; **SMD:** standardised mean difference | | | | | | |
| **GRADE Working Group grades of evidence** **High certainty:** we are very confident that the true effect lies close to that of the estimate of the effect. **Moderate certainty:** we are moderately confident in the effect estimate: the true effect is likely to be close to the estimate of the effect, but there is a possibility that it is substantially different. **Low certainty:** our confidence in the effect estimate is limited: the true effect may be substantially different from the estimate of the effect. **Very low certainty:** we have very little confidence in the effect estimate: the true effect is likely to be substantially different from the estimate of effect. | | | | | | |
