# Supplementary material for: Assessing the impact of contraceptive use on mental health among women of reproductive age – a systematic review
Source: BMC Pregnancy Childbirth. 2024 May 30;24:396. doi: 10.1186/s12884-024-06587-9 (PMC11137968; doi:10.1186/s12884-024-06587-9)
Supplement: Supplementary file 4 — Supplementary Material 4 [file 12884_2024_6587_MOESM4_ESM.docx]

**Appendix 2**


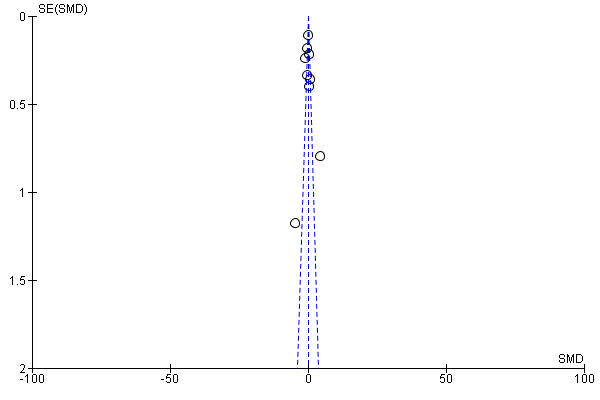


**Figure S1. Risk of publication bias in depression and OCP use**


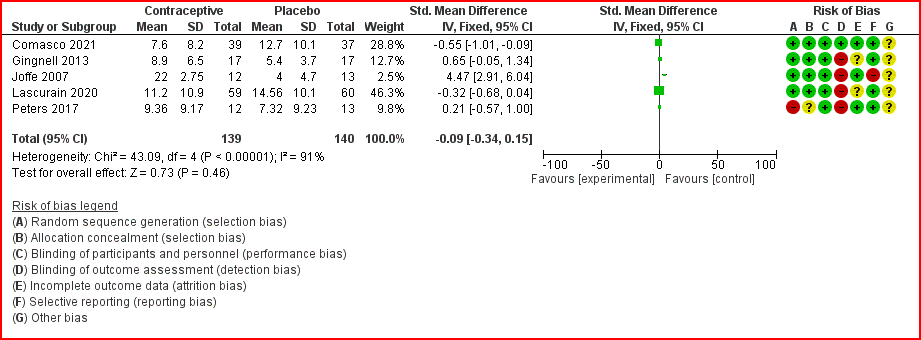


**Figure S2. Use of oral contraceptives versus non-use for the continuous outcome (Montgomery-Asberg Depression Rating Scale) of depression in women with previous mental disorders for five studies**


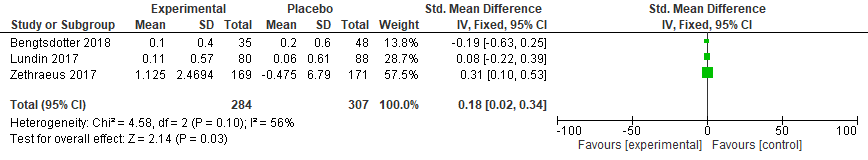


**Figure S3. Forest plot on depression and OCP use for women without previous mental disorders in three studies**


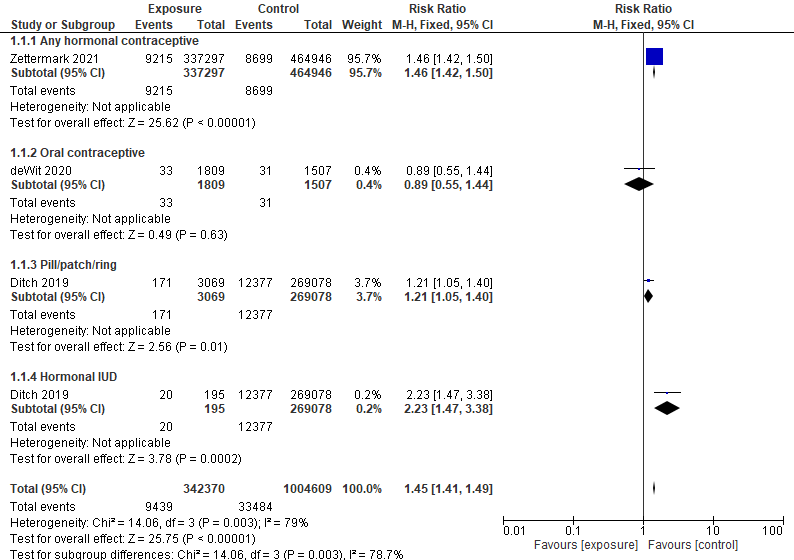


**Figure S4. Use of all hormonal contraceptives versus non-use for the dichotomous outcome of antidepressant use in women without previous mental disorders on general women**


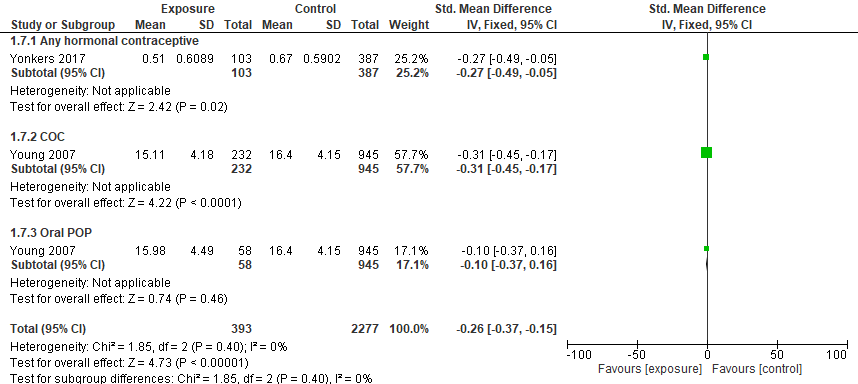


**Figure S5. Use of all hormonal contraceptives versus non-use for the continuous outcome of depression in women with previous mental disorders**


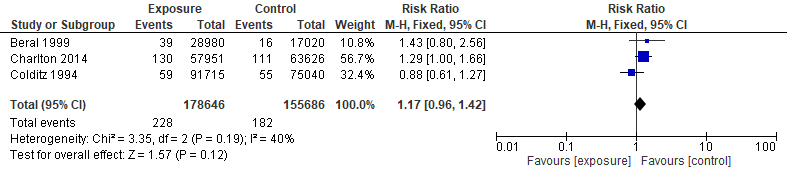


**Figure S6. Use of oral contraceptives use versus non-use for the dichotomous outcome of suicide in general women.**
